# Minor influence of climbing hall characteristics on rubber-derived compound contamination highlights a need for material-level solutions

**DOI:** 10.1039/d5em00812c

**Published:** 2026-01-06

**Authors:** Anya Sherman, Laura Lotteraner, Leah K. Maruschka, Thilo Hofmann

**Affiliations:** a University of Vienna, Centre for Microbiology and Environmental Systems Science, Environmental Geosciences 1090 Vienna Austria thilo.hofmann@univie.ac.at; b University of Vienna, Faculty of Computer Science, Visualization and Data Analysis 1090 Vienna Austria; c University of Vienna, Faculty of Computer Science, Doctoral School Computer Science 1090 Vienna Austria

## Abstract

Climbing shoe abrasion generates fine rubber particles, leading to elevated concentrations of rubber-derived compounds (RDCs) in airborne particulate matter and settled dust of indoor climbing halls, in some cases comparable to levels measured near high-traffic roads. Indoor climbing halls therefore represent a hotspot of RDC exposure for visitors and employees. While the health implications remain uncertain, several RDCs present in climbing halls have demonstrated toxicity *in vitro* and in animal studies. Previous work, limited to a small number of facilities, left open whether climbing hall characteristics can mitigate RDC contamination. Here, we analyzed more than 200 samples of settled dust and foothold powder (abrasion material) collected from 41 climbing halls across 10 countries. RDCs were detected in every sample, confirming their ubiquity. Unsupervised analyses (hierarchical clustering, principal component analysis) revealed distinct patterns in concentrations and profiles, but supervised approaches (redundancy analysis, partial least squares, univariate correlations) showed only weak associations with hall characteristics. These results demonstrate that hall design and operation exert only a minor influence on RDC levels, underscoring that effective mitigation will require material-level solutions, specifically safe and sustainable-by-design (SSbD) innovations in the material used in climbing shoe soles to replace substances of concern with safer alternatives.

## Abstract 2

Rubber-derived compounds are ubiquitous in climbing halls. Climbing hall design and operational practice explain little variability in rubber-derived compound levels, emphasizing that mitigation needs to occur at the product design level.

## Graphical abstract

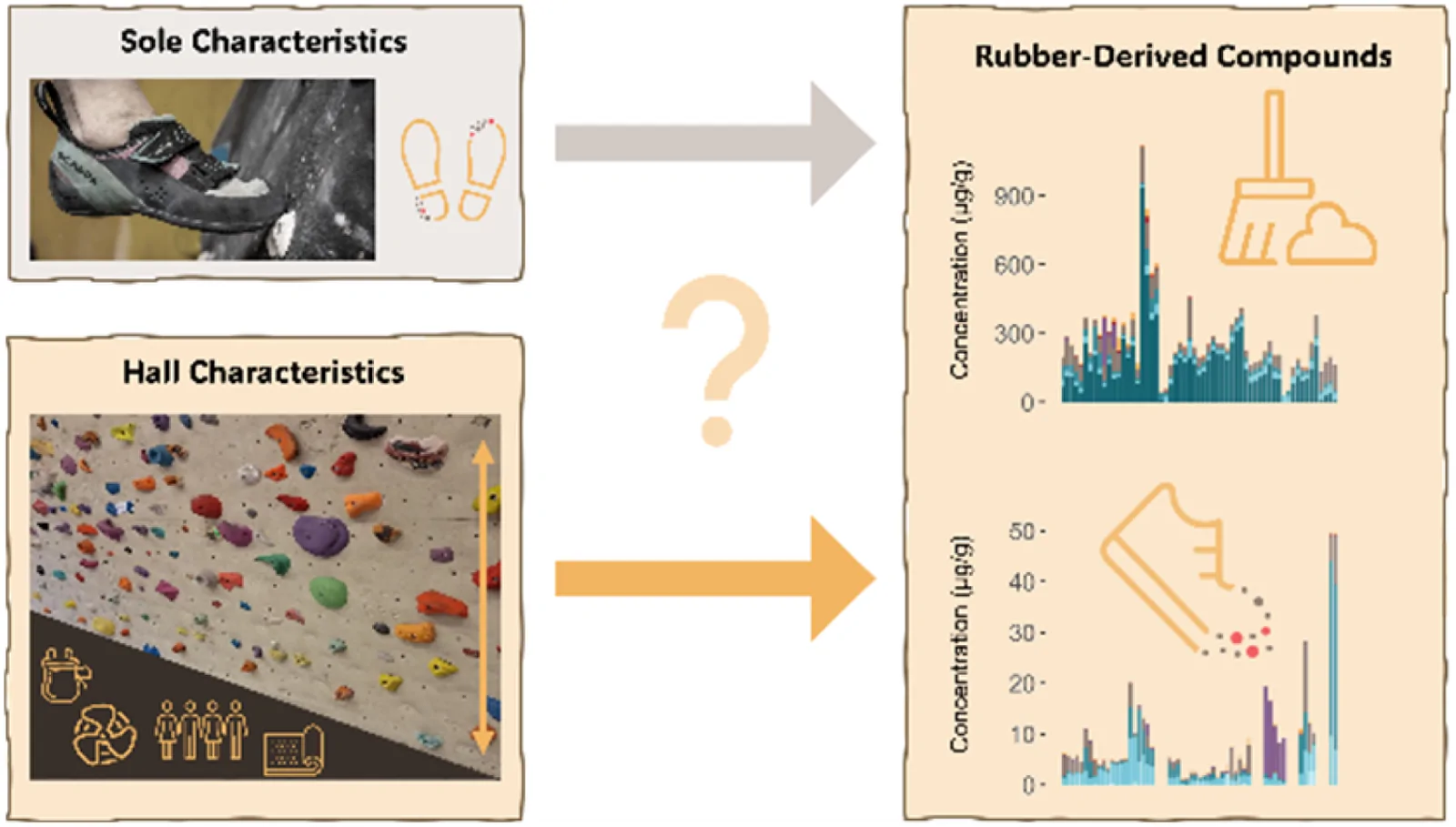

**Environmental significance**

Rubber-derived compounds are a contaminant class of high concern due to their ubiquity in the environment, demonstrated ecotoxicity, and recent detection in multiple human biomonitoring studies. Due to the abrasion of specialized rubber climbing shoe soles, indoor climbing halls are a hotspot of human exposure to rubber-derived compounds. This work demonstrates the ubiquity of rubber-derived compounds in the specific environment of indoor climbing halls, by analyzing over 200 dust and foothold powder samples from 40 halls across ten countries. Weak associations between climbing hall characteristics (building design and operation) and contamination suggest that exposure mitigation requires safe-and-sustainable by design material solutions rather than facility-level interventions.

## 1 Introduction

In indoor climbing halls, abrasion of climbing shoe soles generates fine rubber particles, some of which become airborne, and can be inhaled.^1^ This is of concern to the growing number of people participating in indoor climbing (1.5% in the UK, 4.4% in the US with up to 35 million participants globally^2–4^), and to climbing hall employees who likely represent a highly exposed subpopulation. The manufacturing of climbing shoe soles and other rubber products such as tires that demand elasticity, durability, and grip, requires many organic and inorganic chemical additives. Crosslinkers, such as hexamethoxymethylmelamine (HMMM), are needed to link rubber polymers during the process of vulcanization. Vulcanization accelerators are added to reduce the time and energy needed for the crosslinking process. Some commonly used vulcanization accelerators are benzothiazole and phenyl-guanidine derivatives, such as 2-mercaptobenzothiazole (2SH-BTZ) and 1,3′-diphenylguanidine (DPG).^5^ Another major class of rubber additives are antioxidants, which are added to prevent oxidative degradation of the rubber polymer. Most common in rubber are *p*-phenylenediamine compounds, especially *N*-(1,3-dimethylbutyl)-*N*′-phenyl-*p*-phenylenediamine (6PPD).^6^ In addition to intentionally added compounds, several transformation products are formed during rubber production or use. For example, 6PPD and other *p*-phenylenediamine derivatives form many transformation products, including quinone derivatives, which are in some cases more toxic than the parent compounds.^7,8^ 2SH-BTZ transforms readily to hydroxybenzothiazole (2OH-BTZ) and benzothiazole (BTZ).^9,10^ All of these rubber-derived compounds (RDCs), including parent compounds and transformation products, have been previously detected in climbing halls. Freshly generated rubber abrasion powder collected from footholds (foothold powder) has higher relative quantities of parent compounds. In the finest fraction of these rubber particles, which become airborne, exposure to reactive species such as ozone, hydroxyl radicals, and NO_*X*_ drives a shift to higher relative quantities of transformation products, which dominate the rubber-derived compound profile of airborne particulate matter and settled dust.^1^

In general, rubber-derived compounds are not chemically bound to the polymer, but diffuse within the rubber matrix and into the surrounding environment.^5^ Rubber particles are therefore a source of chemicals of concern to the environment and to humans, where they may be of toxicological concern. For example, leaching of 6PPD-quinone from tire and road wear particles into urban rivers has led to massive fish die-offs,^11^ and various rubber-derived compounds have been shown to leach from rubber particles in simulated biological fluids.^12,13^*In vitro* and rodent studies have demonstrated mammalian hepatoxicity, neurotoxicity, reproductive toxicity, as well as toxicity to the respiratory system of RDCs, especially for 6PPD and 6PPD-quinone.^14–19^ DPG is listed by ECHA as a potential respiratory irritant and potential fertility disruptor.^20^ Benzothiazoles seem to have very low toxicity upon inhalation,^21^ but are endocrine disruptors^22^ and have been linked to cancer in an epidemiological study.^23^

Given that tires are the largest source of rubber-derived compounds into the environment,^24^ research has largely focused on understanding the factors that influence both the generation^25–27^ and the distribution^28,29^ of tire-wear particles in the environment. Our recent work has demonstrated that indoor air quality is an important consideration in indoor climbing halls, where employees and visitors can be exposed to RDCs at levels comparable to those found near high-traffic roads, so similar mitigation-focused research must be conducted for climbing shoes. Climbing shoe soles are a relevant source of RDCs in these environments, but it is unclear to what extent climbing hall characteristics may influence the generation and distribution of rubber particles and associated rubber-derived compounds. In our recent study, we observed substantial variation in RDC concentrations in foothold powder, airborne particulate matter, and settled dust from different climbing halls. However, that study was limited to ten halls, leaving open the questions of whether the reported values are representative of global RDC contamination in climbing halls, and whether the observed variation can be attributed to specific climbing hall characteristics.^1^

To address these questions, we analyzed absolute concentrations and compound profiles of 14 rubber-derived compounds (RDCs) in 119 foothold powder and 120 settled dust samples from 41 climbing halls with varying characteristics from 10 countries. Foothold powder represents the immediate source of RDC contamination in climbing halls, namely abrasion from climbing shoes. Footholds themselves do not contribute to foothold powder since they are made of polyester or polyurethane, do not abrade substantially, and are not a source of rubber-derived compounds.^1^ Settled dust represents a composite sample of airborne particulate matter over time. Although airborne particulate matter concentrations in climbing halls vary temporally, with the highest degree of aerosolization during peak hours,^30^ settled dust is a commonly sampled matrix^31–33^ because its concentrations correlate with airborne levels of organic pollutants.^34^

We evaluated the effect of ten climbing hall characteristics on RDC concentrations and compositions: hall type (bouldering *vs.* rope halls), chalk policy (use of magnesium carbonate powder *vs.* substitution with liquid chalk), mat type (smooth surface *vs.* fiber mats), wall type (friction-coated *vs.* uncoated walls), ventilation mode (active systems *vs.* natural ventilation through windows or doors), hall age, number of weekly visitors, total wall surface area available for climbing, route duration (time footholds remain on the wall before replacement), and visitor density (weekly visitors normalized by wall area). We expected that source generation (RDC concentrations in foothold powder) would be most strongly related to hall type, wall type, visitor numbers, and wall surface area, whereas distribution (RDC concentrations in settled dust) would be influenced by ventilation, chalk policy, mat type, wall surface area, and route duration. The goal of this study was to determine the relative importance of climbing hall characteristics in shaping RDC concentrations and profiles, and thus their potential relevance for human exposure.

## 2 Experimental

### 2.1 Sample collection

We analyzed 119 samples of foothold powder (abrasion material) and 120 samples of settled dust collected from 41 climbing halls in 10 countries (Austria, Estonia, France, Germany, Ireland, the Netherlands, Norway, Spain, Switzerland, USA). Samples from Halls 01 to 11 were collected and analyzed by the authors as part of a previous study.^1^ All other samples were collected by volunteers who received sampling instructions (see SI.2 in the SI) and a kit containing 7 cleaned and labeled glass vials, a metal spatula for collecting samples, and dust-free tissues for cleaning the spatula. Two types of samples were collected in triplicate from each climbing hall. Foothold powder was collected from the clefts of footholds and mostly consists of abrasion powder generated from contact between climbing shoes and the holds. Settled dust was collected from behind or above climbing walls, and from other areas of the gym where particles must have been airborne before deposition, rather than falling directly from the wall. Sampling locations were documented with photos, and volunteers filled out a survey describing the climbing halls where samples were collected. All samples and surveys were returned to Vienna *via* post for extraction and analysis. The survey results are provided in survey.csv in the SI.

### 2.2 Sample extraction and analysis

Samples were extracted and analyzed *via* our previously published method.^1^ Briefly, 50 mg of sample were added to a pre-cleaned accelerated solvent extraction (ASE) cell, and extracted *via* three static cycles of 5 minutes at 80 °C using 100% acetonitrile as solvent. The extracts were concentrated to approximately 0.5 ml under N_2_ blowdown, then reconstituted to 2 ml with acetonitrile, and filtered through 0.45 µm nylon filters before analysis. Surrogate standards (BTZ-d4 and 6PPDQ-d5) were spiked before extraction for a final nominal concentration of 400 ng ml^−1^. Samples were extracted in batches of 20 samples along with two method blanks. Contamination in the method blanks was monitored (Table SI.3.1 in the SI) and subtracted from concentrations measured in samples of that batch. Samples were analyzed with UPLC-MS/MS (Agilent 1290 Infinity II, Agilent 6470) with compound specific details provided in Table SI.4.1 in the SI. Method details including limits of quantification and recovery data are provided in Tables SI.5.1 and SI.6.1 in the SI. To enable high-throughput analysis of a large sample set, 14 rubber-derived compounds were prioritized for analysis based on prior detection in climbing halls,^1^ detection in human biomonitoring studies^35–38^ or of potential toxicologic concern,^39^ chemical diversity, and availability of analytical standards. Rubber-derived compounds analyzed were: benzothiazole (BTZ), 2-hydroxybenzothiazole (2OH-BTZ), 2-aminobenzothiazole (2NH-BTZ), 2-mercaptobenzothiazole (2SH-BTZ), 1,3-diphenylguanidine (DPG), hexa(methoxymethyl)melamine (HMMM), the phenylenediamine compounds 6PPD, IPPD, CPPD and DPPD and their associated quinones, 6PPDQ, IPPDQ, CPPDQ and DPPDQ.

### 2.3 Data preparation

Outlier samples are defined by a multivariate concentration profile inconsistent with the dominant structure of the dataset. While these samples may reflect real but uncommon conditions affecting compound concentrations, their atypical behavior means they would disproportionately drive the statistical results, so they were excluded from the modeling. Outlier samples were identified for foothold powder and settled dust samples separately. Samples with a squared Mahalanobis distance, computed by the Generalized S-Estimate^40^ from the R package GSE,^41^ above the 90% quantile were flagged as outliers. A single outlier sample in a hall was assumed to be due to a random irregularity in the sample and removed from the dataset. If multiple samples from the same hall were flagged as outliers, the hall was assumed to have a unique contamination pattern, and these samples were retained. Photos from outlier sample locations did not indicate sampling errors.

In both the foothold powder and settled dust samples values below the LOQ were substituted with a random number between 0 and the LOQ. Considering both the moderate degree of censoring (see Fig. 2, and Tables SI.7.1, SI.7.2 in the SI) and variable LOQs between batches this has been determined the optimal method for handling <LOQ values for the calculation of summary statistics and multivariate analyses.^42,43^

To assess the composition of rubber particles within the samples regardless of dilution effects inherent in absolute concentrations, all individual concentrations were normalized to the sum of all detected rubber-derived compounds in each sample.

Outliers in the quantitative climbing hall characteristics from the survey dataset were assumed to be due to reporting errors. Based on the assumption that a single incorrectly reported climbing hall characteristic in a survey did not influence the correctness of the other characteristics, univariate outliers *X* were identified based on the interquartile range of each climbing hall characteristic, by *X* > *Q*_75_ + 1.5 × (*Q*_75_ − *Q*_25_). All raw data (before <LOQ substitution, outlier removal, and normalization) are provided in smp.csv in the SI. The survey results are provided in survey.csv in the SI.

### 2.4 Statistical analysis

Statistical analysis was performed in R 4.1.2 ^44^ and done separately for foothold powder and settled dust concentrations and compositions. Due to the non-euclidean nature of compositional data, methods were adapted by applying isometric log-ratio (ILR) transformation from the R package compositions^45^ and using Aitchison instead of Euclidean distance^46^ as detailed below.

To identify the compounds most relevant for the variation between samples, principal component analysis (PCA) was applied to concentration data, and principal coordinate analysis (PCoA), using the R package ape,^47^ was applied to composition data.

Hierarchical clustering, specifically agglomerative clustering,^48^ was used to identify patterns in the samples across halls. Clusters were defined using the Euclidean distance for concentrations and the Aitchison distance for composition, combined with the ward similarity. The ideal number of clusters for each sample type was determined by a combined evaluation of the resulting dendrograms (see Section SI.8 in the SI) and PCA/PCoA plots colored by clusters.

The percentage of variance in the multivariate dataset of rubber-derived compounds that the multivariate dataset of climbing hall characteristics can explain was assessed using redundancy analysis (RDA) for concentrations and distance-based redundancy analysis (dbRDA) with the Aitchison distance for compositions, both from the R package vegan.^49^ RDA does not accept missing values, so only samples from the 56% of halls that reported all characteristics were included in the analysis: 64 foothold powder and 66 settled dust samples.

Relationships between the multivariate dataset of rubber-derived compounds and single climbing hall characteristics were assessed with partial least squares regression (PLS) for numerical hall characteristics, and partial least squares discriminant analysis (PLS-DA) for categorical hall characteristics, both from the R package mixOmics.^50^ For each hall characteristic, samples from all halls that reported it were included in the analysis, resulting in between 57 and 116 samples of both foothold powder and settled dust samples.

PLS models were evaluated based on *Q*^2^ (where *Q*^2^ = 1 means perfect predictive ability), PLS-DA models based on the relative balanced error rate (BER_rel_ = BER_model_/BER_random_ where BER_rel_ < 1 means a predictive ability better than random and BER_rel_ = 0 means perfect predictive ability). To ensure robust results, the calculation of the random BER was repeated ten times. In PLS models for concentration data, the importance of individual compounds in the model was determined based on the variable importance in projection (VIP) scores. Due to the ILR transformation, VIP scores cannot be interpreted directly for compositional data. Details on the implementation, and all PLS and PLS-DA model scores and VIP scores are provided in Sections SI.9 and SI.10 in the SI.

Relationships between single compounds and single hall characteristics were identified using the Kruskal–Wallis rank sum test^51^ for categorical and Spearman's correlation for numerical hall descriptors. The *p*-values were adjusted for multiple hypothesis testing using the Benjamini & Hochberg correction.

## 3 Results and discussion

### 3.1 Data overview

**Fig. 1:**
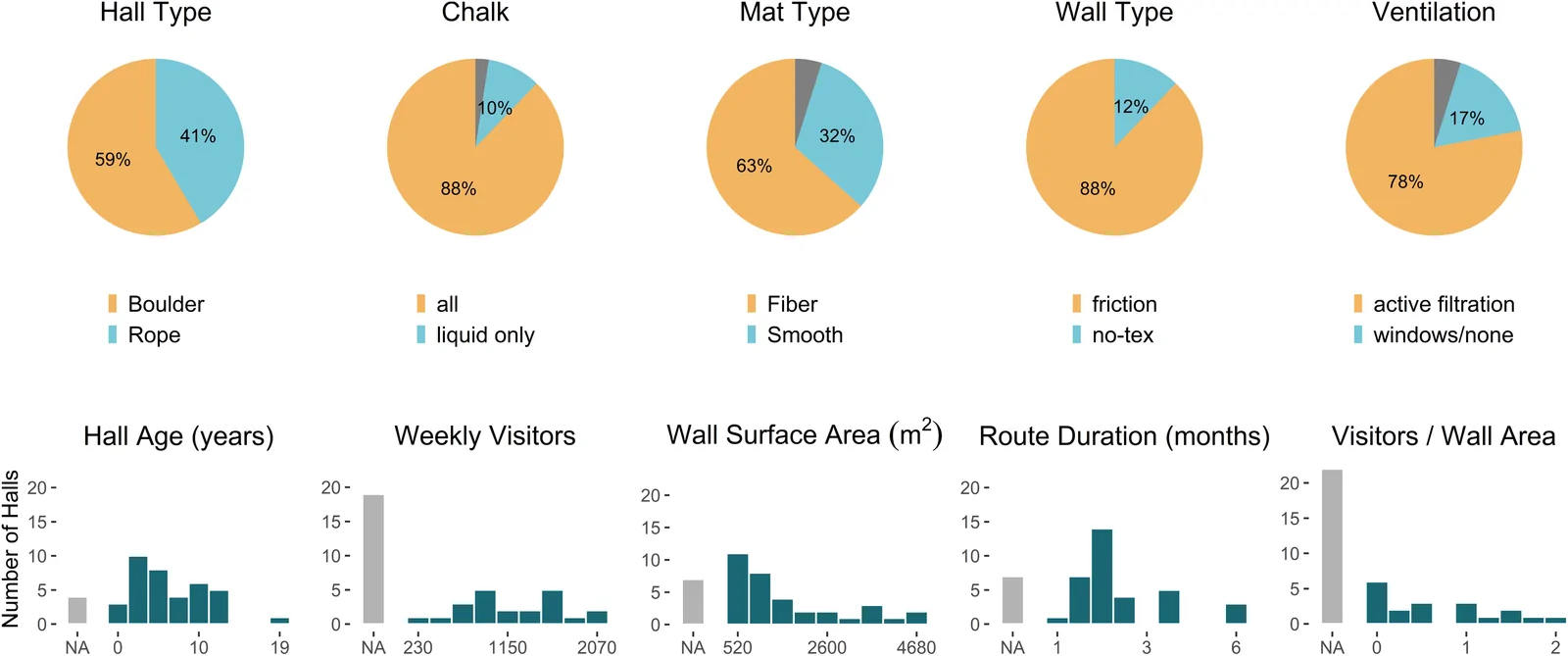
Climbing hall characteristics (outliers excluded).

59% of halls analyzed were bouldering halls, where climbers ascend short (<3 m), more difficult routes before jumping down onto cushioned mats, while 41% were rope halls, where climbers ascend longer routes attached to a rope. Most rope climbing halls also have bouldering areas. 88% of halls permit climbers to apply chalk (MgCO_3_) to their hands to enhance grip, which has been identified as the dominant source of particulate matter in climbing hall air.^30^ Particulate matter levels can be reduced by banning chalk, or permitting only liquid chalk (MgCO_3_ dissolved in ethanol),^52^ and 10% of the halls analyzed in this study permit only liquid chalk. Most halls (63%) have fibrous surface mats, which are expected to retain settled particles. However, 32% had smooth vinyl surfaces, from which particles may be re-aerosolized when impacted by falling climbers. 88% of halls had friction-coated climbing walls, while 12% had smooth “no-tex” walls, which is believed to reduce the abrasion of climbing shoes. 78% of climbing halls had active ventilation systems, while 17% had no ventilation, relying only on windows and doors, see Fig. 1.

The aforementioned distribution of climbing hall characteristics was used for PLS-DA models and univariate analyses, however, excluding halls with missing characteristics for RDA reduced the total number of halls to 18, with 50% rope and 50% boulder halls, 94% halls allowing chalk and 6% only allowing liquid chalk, 67% fiber and 33% smooth mats, 83% friction-coated and 17% smooth walls, and 94% halls with ventilation and 6% without.

Climbing halls were between 0 and 27 years old, with the majority (63%) under 10 years. Halls had between 250 and 4800 weekly visitors, although 18 halls did not report visitation numbers. Climbing wall surface area, which is related to hall type and can be used as a proxy for hall size, ranged from 280 to 6875 m^2^. Average route duration (the frequency of climbing hold removal, cleaning, and resetting) ranged from 1 to 12 months, with most halls (56%) resetting routes around every 2 months. The number of weekly visitors per climbing wall surface area, calculated to estimate the climbing activity (and climbing shoe abrasion) relative to the size of the climbing hall, ranged from 0.2 to 2.5 people per m^2^ per week, see Fig. 1.

After excluding halls with missing characteristics for RDA, the numbers changed to hall age between 1 and 13 years, between 280 and 4500 weekly visitors, wall surface area between 280 and 4500 m^2^, and between 0.2 and 2.5 visitors per m^2^ per week.

**Fig. 2:**
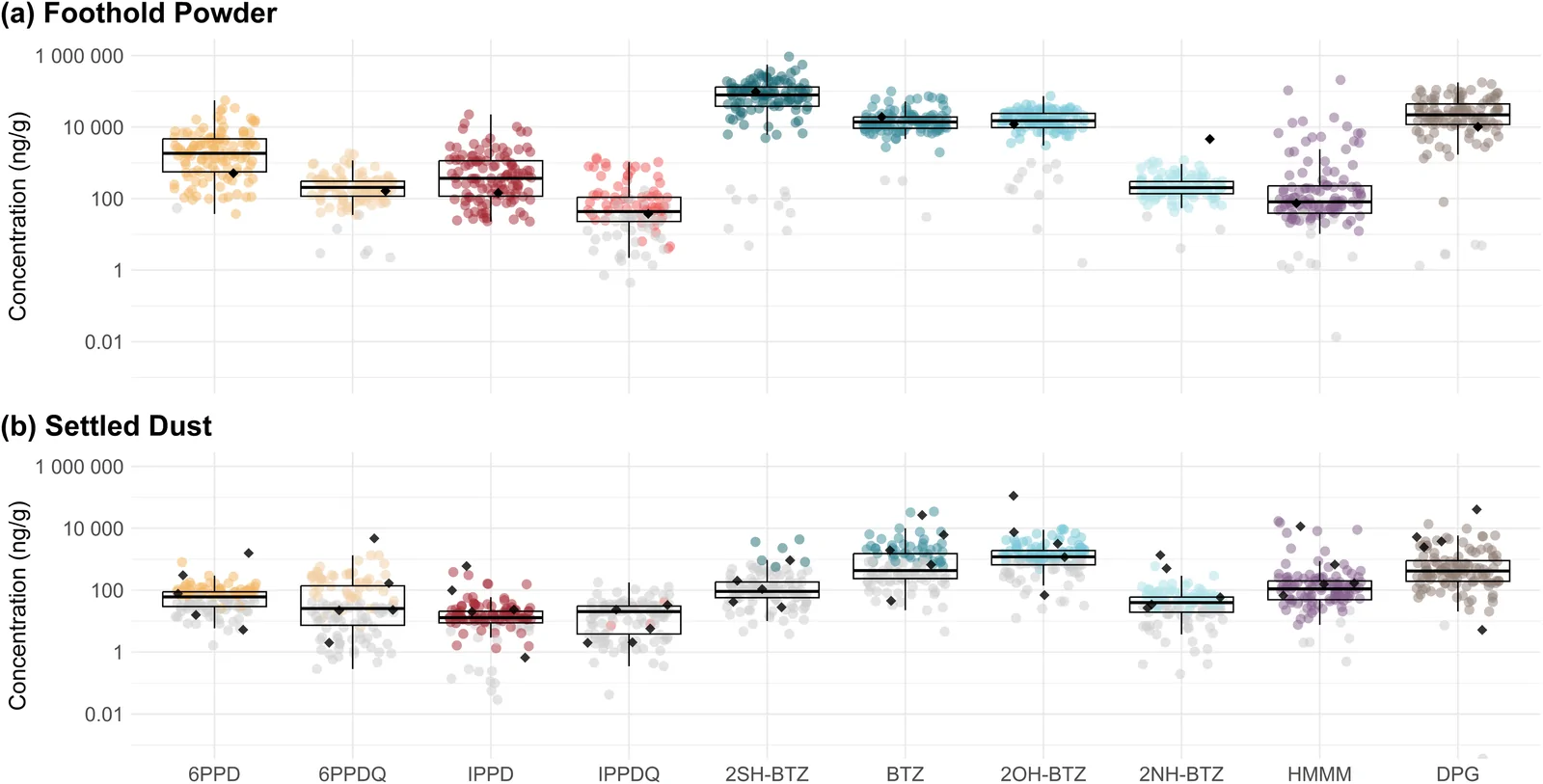
Concentrations of rubber-derived compounds in (a) foothold powder and (b) settled dust. Gray color indicates <LOQ values (for the respective batch) which were substituted by random numbers between 0 and the (respective batch) LOQ for statistical analyses, see Section 2.3. Due to LOQs varying between batches some compounds may have substituted values higher than some measured values. Outlier values that are removed before analysis are displayed in black.

Despite these differences in climbing hall characteristics, rubber-derived compounds were detected in every single foothold powder and settled dust sample, underscoring their ubiquity in indoor climbing halls. Fig. 2 shows the concentrations of RDCs in both foothold powder and settled dust, with randomly substituted values below the respective batch LOQ (see Section 2.3) in gray. Due to LOQs varying between batches some compounds may have substituted values higher than some measured values.

Foothold powder samples are predominantly composed of abraded rubber from climbing shoes, although contributions from chalk and other sources can not be excluded. In foothold powder, the most frequently detected compounds were the antiozonants 6PPD and IPPD, found in 99% and 100% of samples, at mean concentrations of 4400 ng g^−1^ and 117 ng g^−1^, respectively. 6PPD and IPPD are likely the most commonly used *p*-phenylenediamine antiozonants in climbing shoe soles, which is comparable to other rubber products.^6^ Alternative antiozonants CPPD and DPPD were detected in 70% and 24% of foothold powder samples, at lower mean concentrations of 22.5 ng g^−1^ and 99.0 ng g^−1^. This suggests that CPPD and DPPD are likely present in climbing shoe rubber only as impurities or minor components. Generally, PPD concentrations were much lower than those reported in tire particles,^12,53^ which may be due to different material demands (tires are exposed to much higher concentrations of ozone and other reactive oxygen species at the road surface). The PPD concentrations detected were, however, similar to those found in crumb rubber, which is mainly composed of old tires that have already undergone substantial environmental weathering.^53^

PPDs react with ambient reactive oxygen species to form many transformation products, including quinone derivatives.^8,11^ Consequently, 6PPDQ and IPPDQ were frequently (95% and 59% of samples) detected in foothold powder samples, at mean concentrations of 283 ng g^−1^ and 227 ng g^−1^, respectively, which is about one order of magnitude lower than their respective parent compounds. While IPPDQ concentrations in foothold powder are slightly higher than reported in crumb rubber, 6PPDQ concentrations are lower than reported in tire wear and crumb rubber.^53^ CPPDQ and DPPDQ were not detected in any foothold powder samples.

In settled dust, 6PPD was detected in 60% and 6PPDQ in 43% of samples. Unlike in foothold powder, 6PPDQ was detected at higher concentrations (mean 116 ng g^−1^) than 6PPD (mean 73 ng g^−1^), indicating a high extent of transformation. Accordingly, 6PPD concentrations in settled dust were generally at background levels similar to those which have been reported in household dust,^31^ while extensive transformation results in elevated 6PPDQ concentrations compared to other environments.^54,55^ IPPD was also detected in 90% of settled dust samples, at a mean concentration of 24.8 ng g^−1^, while IPPDQ was only detected in 5% of samples, at a mean concentration of 14.5 ng g^−1^. This may indicate that IPPD transformation to IPPDQ proceeds more slowly than 6PPD to 6PPDQ, which has been observed in a previous study of household dust.^55^ IPPD and IPPDQ concentrations in climbing hall dust are higher than those which have been reported in household dust.^55,56^ CPPD, DPPD, and their respective quinones were all detected in less than 10% of settled dust samples. We concluded that these compounds are only minor pollutants in indoor climbing halls, and excluded them from all further statistical analyses.

2SH-BTZ was detected in 92% of foothold powder samples, at the highest concentrations of any rubber-derived compound (mean: 107 000 ng g^−1^), suggesting that it is the primary vulcanization accelerator used in climbing shoe rubber. This is higher than 2SH-BTZ concentrations reported in tires and tire debris by Zhang *et al.*,^57^ but lower than reported by Avagyan *et al.*^58^ Two other benzothiazole derivatives, 2OH-BTZ and BTZ, had similarly high detection frequencies −91% and 95% in foothold powder, albeit at concentrations about one order of magnitude lower than 2SH-BTZ (mean concentrations of 18 800 ng g^−1^ and 18 400 ng g^−1^, respectively). While 2OH-BTZ concentrations are similar to those which have been reported in tires,^57^ BTZ concentrations in tires are slightly higher^57,58^ than those in foothold powder. Both 2OH-BTZ and BTZ are known transformation products of 2SH-BTZ,^9,10^ and while some quantity of these benzothiazoles may also be intentionally added to climbing shoe rubber, their formation in foothold powder due to interactions with reactive oxygen species has been demonstrated.^1^ A fourth benzothiazole derivative, 2NH-BTZ, was detected in 97% of foothold powder samples, at a much lower mean concentration of 260 ng g^−1^, which is lower than reported in tires.^57^ 2NH-BTZ is not a known environmental transformation product of 2SH-BTZ, so 2NH-BTZ is likely added to rubber blends directly, possibly as a minor component of a blend of benzothiazoles. Overall, these data indicate notable differences between benzothiazole concentrations in tires and climbing shoes.

Despite the high detection frequency and concentrations in foothold powder, 2SH-BTZ was detected in only 8% of settled dust samples. BTZ and 2OH-BTZ were detected in 41% and 66% of settled dust samples with mean concentrations of 4000 ng g^−1^ and 3800 ng g^−1^, respectively. This indicates a large extent of transformation from 2SH-BTZ to BTZ and 2OH-BTZ, as was observed for 6PPD and 6PPDQ, and reported previously in climbing halls.^1^ 2NH-BTZ was detected in 56% of settled dust samples at a mean concentration of 122 ng g^−1^. BTZ, 2SH-BTZ and 2NH-BTZ concentrations in climbing hall settled dust are higher than previously reported in indoor dust;^33,59^ 2OH-BTZ concentrations are higher than^33^ or similar to^59^ those reported in indoor dust. This confirms that benzothiazole concentrations in indoor climbing halls generally exceed those in other indoor environments, and implicates a high exposure for climbing hall visitors and employees.

DPG, another vulcanization accelerator, was detected in 96% of foothold powder samples, at a mean concentration of 33.4 µg g^−1^. DPG was the rubber-derived compound present at the second-highest concentrations in foothold powder, but lower than those reported in tire wear particles,^53^ which indicates that DPG may be a less important vulcanization accelerator in climbing shoe rubber compared to tires. However, the concentrations were similar to those found in crumb rubber,^53^ suggesting that the DPG concentrations in foothold powder may have been partly reduced by aging. DPG was also detected in 100% of settled dust samples at a mean concentration of 1070 ng g^−1^, which is higher than or similar to previous reports of DPG in house dust,^32,33,38^ implicating climbing halls as a hotspot for DPG exposure.

HMMM was detected in 90% of foothold powder and 93% of settled dust samples, at mean concentrations of 3700 ng g^−1^ and 655 ng g^−1^, respectively. HMMM is added to rubber as a crosslinking agent but is also used in plastic and metal coatings^60^ and may have alternate sources in some climbing halls.^1^

Out of 119 foothold powder samples, 12 samples from 6 halls were flagged as outliers, including the 11 samples with the highest HMMM concentrations. Only a single outlier, which had a lower HMMM but distinctly higher 2NH-BTZ concentration, was removed for further analysis, see Fig. 2a. Out of 120 settled dust samples, 13 samples from 8 halls were flagged as outliers, including the 6 samples with the highest HMMM concentrations. These samples were from the three halls with the highest HMMM concentrations in foothold powder, indicating an alternative HMMM source in some climbing halls. Five of the 13 outliers, including samples with distinctly higher 6PPD, 6PPDQ and 2OH-BTZ concentrations, were removed for further analysis, see Fig. 2b. While the concentrations of these outlier samples might not look particularly high for the individual compounds, their multivariate structure differs from the majority of the data.^40^

### 3.2 Trends in concentrations and composition

RDC concentrations are expected to vary between samples within a hall, depending on sampling time and location. Groups of samples with similar concentrations or compositions were identified using hierarchical clustering, and the results were confirmed using PCA and PCoA. To verify that these groups were not random, but related to the climbing halls, the percentage of halls with all samples in the same cluster was compared with the percentage expected for a random assignment of clusters, accounting for differences in cluster size.

Two clusters for foothold powder concentrations and four clusters for foothold powder compositions could be identified. 68% of halls had all three samples in the same cluster for both concentration and composition data, compared to 25% and 42% respectively, expected for random cluster assignment. For settled dust concentrations and compositions, six and five clusters were identified, with all three samples of a hall being in the same cluster for 61% (compared to 16% for random assignment) and 49% (compared to 8% for random assignment), of all halls. These results indicate that differences between samples are not random, but related to differences between halls, which could be potentially ascribed to climbing hall characteristics.

**Fig. 3:**
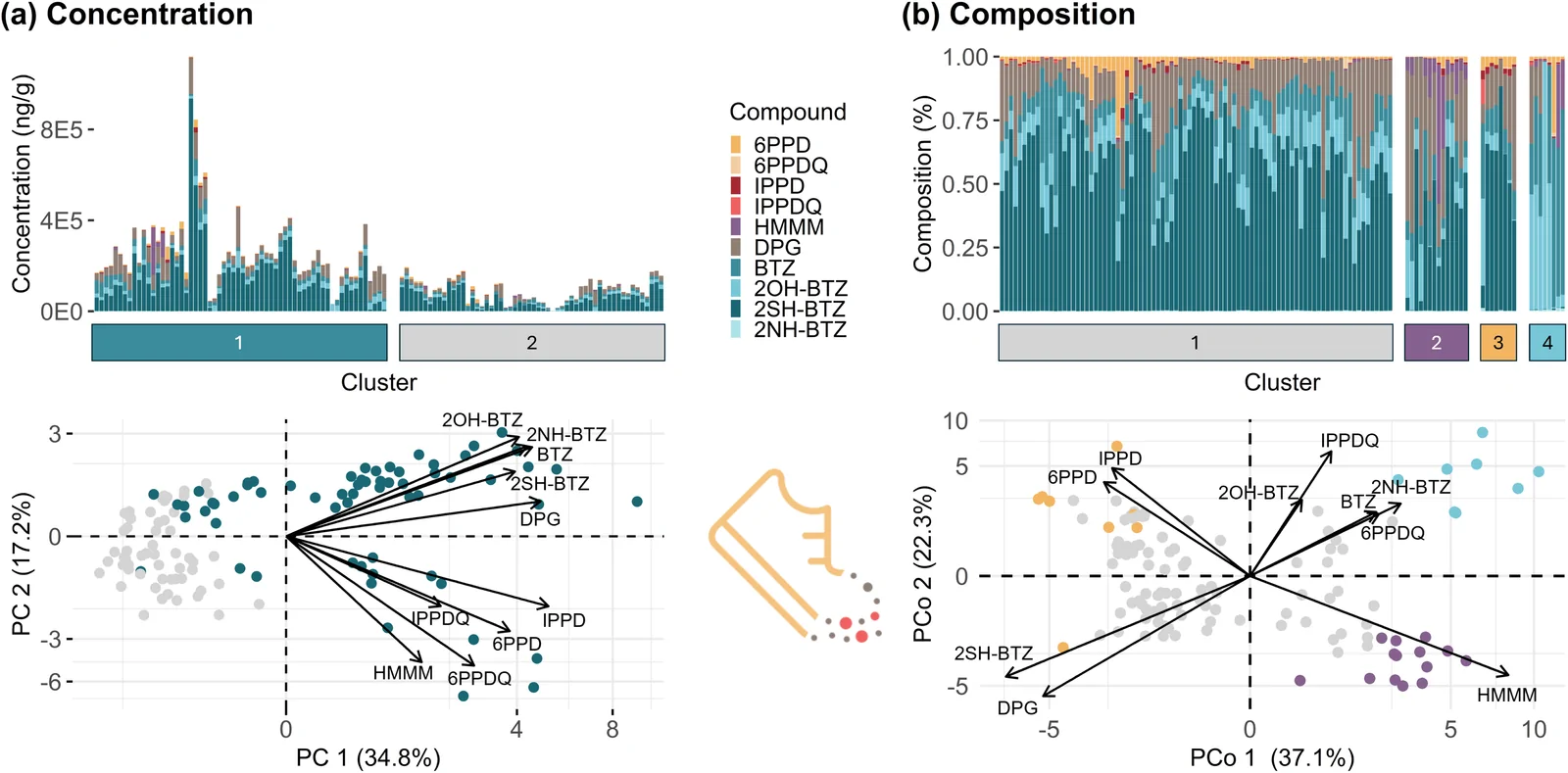
Concentrations (a) and compositions (b) of rubber-derived compounds in foothold powder samples ordered by the respective clusters, combined with the first two principal components (a) and principal coordinates (b), ordered and colored by the clusters. In both cases, the first two principal components account for around half of the total variation in the data. The bars and points correspond to individual samples (not averages of triplicates). In combination, these figures allow the interpretation of the clusters in relation to individual compounds.

The foothold powder samples are split into two clusters according to their absolute concentrations, with a high-concentration (*i.e.*, low-dilution) and a low-concentration (*i.e.*, high-dilution) cluster, see Fig. 3a. Foothold powder can be diluted with *e.g.* chalk, if footholds are also used as handholds. Also, ambient particulate matter can accumulate on the footholds in addition to rubber abrasion.

According to their compositions (see Fig. 3b), most foothold powder samples fall into cluster 1, with a generic foothold powder composition dominated by 2SH-BTZ, and substantial contributions of 2OH-BTZ, BTZ, and DPG. Most samples also contain 6PPD and IPPD, although the amount varies. Cluster 2 includes samples with the highest HMMM contributions. Cluster 3 can not be directly attributed to any individual compounds, though all samples contain 6PPD and IPPD. Samples in cluster 4 contain (almost) no 2SH-BTZ but more 2OH-BTZ than other samples, indicating these samples are older than the others.

**Fig. 4:**
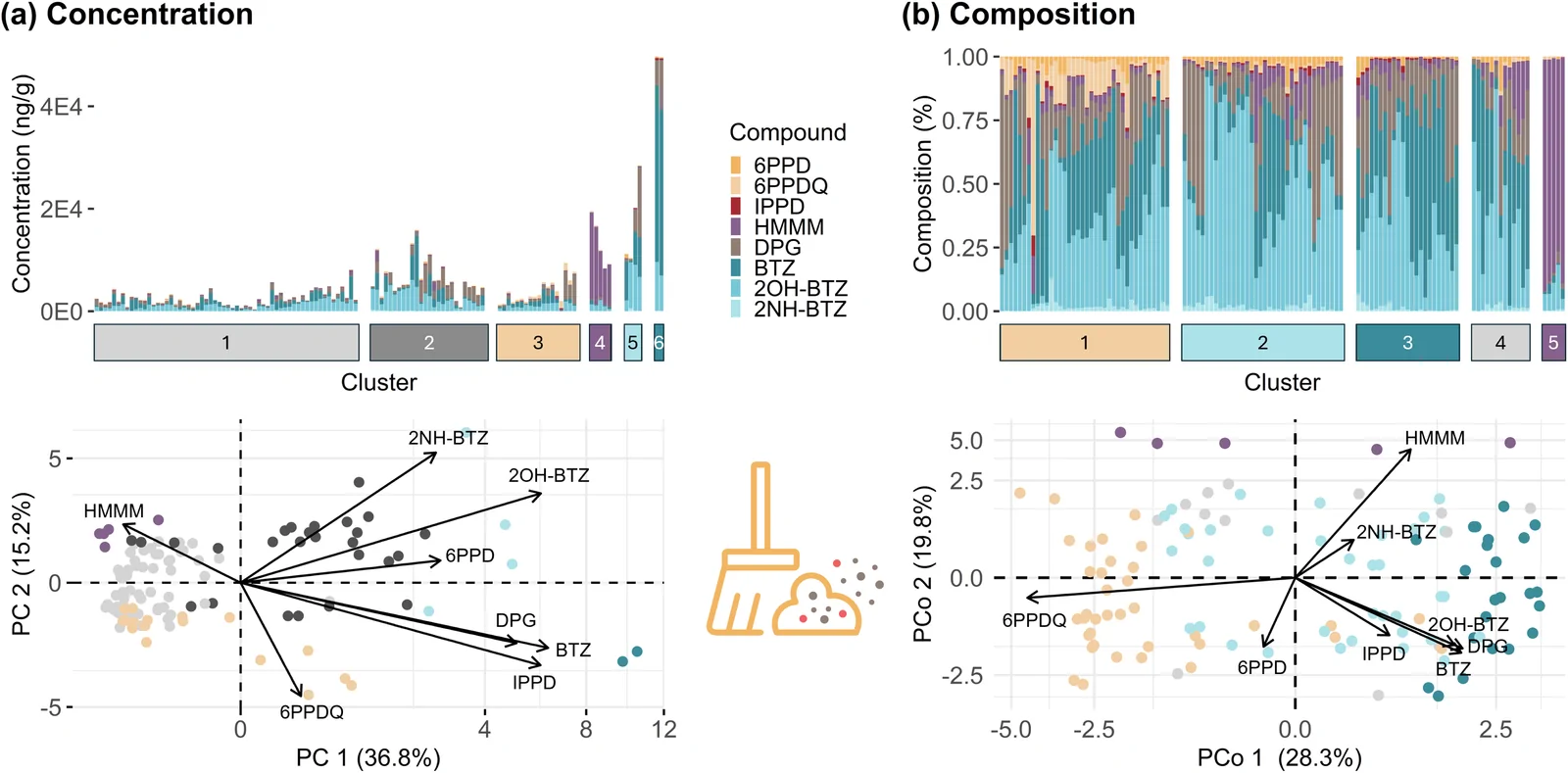
Concentrations (a) and compositions (b) of rubber-derived compounds in settled dust samples ordered by the respective clusters, combined with the first two principal components (a) and principal coordinates (b), ordered and colored by the clusters. In both cases, the first two principal components account for around half of the total variation in the data. The bars and points correspond to individual samples (not averages of triplicates). In combination, these figures allow the interpretation of the clusters in relation to individual compounds.

Settled dust samples are also split according to their absolute concentration (see Fig. 4a), with the general low-concentration (*i.e.*, high-dilution) cluster 1 and the general higher-concentration (*i.e.*, lower-dilution) cluster 2. As foothold powder, settled dust can be diluted with *e.g.* chalk, but even more with ambient particulate matter. An additional dilution by polymeric particles released by the abrasion of ropes in climbing halls can likely be neglected as climbing ropes, in contrast to climbing shoes, are designed for minimal abrasion. However, four clusters relate to individual compounds. Cluster 3 has generally lower concentrations (*i.e.*, higher dilution), with higher 6PPDQ concentrations. Cluster 4 is dominated by HMMM, with all other compounds present in low concentrations. Cluster 5 contains the samples with the highest DPG and 2NH-BTZ concentrations. The cumulative concentration in the two samples of cluster 6 is much higher than in all other samples and is dominated by very high BTZ concentrations.

The most notable distinction between foothold powder and settled dust compositions is the absence of 2SH-BTZ in settled dust, and the relatively higher contributions of BTZ and 2OH-BTZ. This can be attributed to the rapid transformation of 2SH-BTZ to these two compounds in airborne rubber particles.^1^ Clustering by compositions showed that the most distinct cluster 5 is dominated by HMMM and corresponds directly to cluster 4 in the settled dust absolute concentrations (see Fig. 4b). The other clusters can not be easily attributed to any specific compound, but some trends are visible. Cluster 1 has the highest 6PPDQ levels and cluster 2 has the highest 2OH-BTZ and 2NH-BTZ levels while also having lower BTZ levels compared to the other clusters.

The two samples assigned to cluster 6 based on their rubber-derived compound concentrations in settled dust have distinctly higher cumulative concentrations than all other samples. These were considered additional outliers and removed for further analysis. The samples assigned to cluster 4 based on concentrations in settled dust had HMMM concentrations much exceeding all other samples (7070–17 000 ng g^−1^ in cluster 4 *versus* 0.49–2220 ng g^−1^ in all other clusters). Considering that the maximum HMMM concentration reported in climbing shoe soles (*n* = 30) was 429 ng g^−1^,^1^ there is almost certainly an alternative HMMM source in these climbing halls. As an alternative HMMM source confounds statistical analyses intended to identify relationships between RDCs stemming from climbing shoe sole rubber and climbing hall characteristics, further statistical analyses were conducted with and without samples in Cluster 4 (SI.13).

### 3.3 Relationships between rubber-derived compounds and climbing hall characteristics

Foothold powder and settled dust concentrations and compositions were analyzed for relationships with the climbing hall characteristics using three different approaches: RDA (and distance-based RDA for compositional data) analyzes relationships between all climbing hall characteristics and all rubber-derived compounds, and is the most comprehensive method to evaluate multivariate relationships in our dataset. For each RDA (or dbRDA) model, both the total variance in rubber-derived compounds that can be explained by the combination of all climbing hall characteristics and the marginal contributions of each characteristic are reported. However, due to the major limitation that RDA can not handle missing values, the analysis could only be conducted on samples from halls for which all characteristics were reported, which led to the exclusion of 46% of foothold powder and 45% of settled dust samples (44% of halls). PLS (and PLS-DA) analyze relationships between a single climbing hall characteristic and all rubber-derived compounds, thus including a larger number of halls in the analysis than (db)RDA. Additionally, we analyzed univariate relationships between single climbing hall characteristics and single rubber-derived compounds.

For each approach individually, climbing hall characteristics were considered relevant according to manually-defined, rather low thresholds, as there are no standardized thresholds for these analyses to the best of our knowledge. Accordingly, climbing hall characteristics are considered relevant if they explain at least 5% of variation in (db)RDA, have *Q*^2^ > 0.1 in PLS or BER_rel_ < 0.9 in PLS-DA models, or have significant univariate relationships with at least three rubber-derived compounds. The different approaches work with different subsets of the entire dataset, and test for different types of relationships. Considering the potential sensitivity to methods and data selection,^61^ for robust results we only discuss climbing hall characteristics in depth that were identified as relevant by at least two of these three statistical methods (see Table 1). Detailed results for PLS(-DA), univariate correlations, and (db)RDA are presented in Sections SI.10, SI.11, and SI.12, respectively, in the SI.

**Table 1:** Combined results of (db)RDA, PLS(-DA) and univariate correlations for foothold powder and settled dust concentrations and compositions. Numbers in brackets are the marginal contribution to the overall variation for RDA and dbRDA, *Q*^2^ for PLS models, BER_rel_ for PLS-DA models, and the number of compounds with a significant relationship in the univariate case. Hall characteristics selected by at least two different models and the corresponding model scores are highlighted in bold

	Foothold powder		Settled dust	
	Concentrations	Compositions	Concentrations	Compositions
**Chalk policy**		**dbRDA (0.05)**		
	**PLS-DA (0.88)**	**PLS-DA (0.51)**		
	**Univariate (4)**	**Univariate (3)**		
**Hall age**	**RDA (0.05)**			
	**PLS (0.16)**			
	**Univariate (3)**			
**Hall type**	PLS-DA (0.70)	**PLS-DA (0.57)**	RDA (0.06)	PLS-DA (0.70)
		**Univariate (5)**		
**Route duration**	PLS (0.13)	**PLS (0.17)**	PLS (0.17)	PLS (0.28)
		**Univariate (3)**		
**Visitors/wall area**		**dbRDA (0.05)**	**RDA (0.09)**	
			**PLS (0.43)**	**PLS (0.56)**
		**Univariate (3)**		**Univariate (3)**
**Wall surface area**		**dbRDA (0.08)**		
	PLS (0.11)	**PLS (0.14)**	PLS (0.16)	**PLS (0.31)**
		**Univariate (3)**		**Univariate (3)**
Mat type	PLS-DA (0.84)	PLS-DA (0.76)		
Ventilation			RDA (0.07)	
Wall type	Univariate (6)	Univariate (3)		dbRDA (0.05)
Weekly visitors	Univariate (3)	dbRDA (0.06)	PLS (0.22)	PLS (0.25)

#### 3.3.1 Foothold powder concentrations

RDA revealed that only 26% of the variation in foothold powder concentrations of rubber-derived compounds could be explained by a combination of all climbing hall characteristics. Hall age was a significant predictor identified by RDA (5% of variation), PLS (*Q*^2^ = 0.16), and univariate analyses, with the effect driven by benzothiazole concentrations (PLS VIP scores >1 and univariate correlations *p* < 0.05). Older halls have slightly higher concentrations of 2OH-BTZ, BTZ, and 2NH-BTZ, while other compounds did not show significant differences. BTZ and 2OH-BTZ are transformation products of 2SH-BTZ, which should increase in concentration with foothold powder sample age – these data suggest that foothold powder is generally older in older halls. 2NH-BTZ is not a major transformation product of 2SH-BTZ. Chalk was also a significant predictor of foothold powder concentrations identified by PLS-DA (BER_rel_ = 0.58) and univariate analyses: almost all compounds had higher concentrations in foothold powder from halls where only liquid chalk is used (although the difference was only statistically significant for 6PPD, 6PPDQ, IPPD and IPPDQ). This is due to less dilution of foothold powder with chalk.

#### 3.3.2 Foothold powder compositions

Similar to the absolute concentrations, climbing hall characteristics explained only 24% of the variation in rubber-derived compounds compositions of foothold powder according to the dbRDA. However, compared to the absolute concentrations, variation in composition was explained by fewer climbing hall characteristics that each had slightly higher contributions to total variation. Chalk was identified by all three statistical methods to be a determining predictor of rubber-derived compound compositions in foothold powder (5% of variation in dbRDA, BER_rel_ = 0.51 in PLS-DA): 6PPD and IPPD were higher in halls using only liquid chalk, while 2OH-BTZ was lower. Although not statistically significant, 2SH-BTZ and 2NH-BTZ followed the same trends as 2OH-BTZ, and 6PPDQ and IPPDQ followed the same trends as 6PPD and IPPD. The reasons for this compound-class specific trend are unclear, however, the results should be interpreted with caution, as chalk is the climbing hall characteristic with the most uneven class distribution (Fig. 1). Hall type was identified to be relevant by PLS-DA (BER_rel_ = 0.57) and univariate analyses. 2SH-BTZ represented a higher fraction of total rubber-derived compounds in bouldering halls, while BTZ, 2NH-BTZ, HMMM, and 6PPDQ represented higher fractions in rope halls. In rope halls, climbing routes are longer with many more footholds, while in bouldering halls, climbing activity is concentrated on a few footholds per route. In addition, bouldering moves are generally more intense and require more intensive contact between the climbing shoes and the footholds. For both reasons, there is a more pronounced generation of fresh rubber abrasion in the foothold powder of bouldering halls, explaining the higher fraction of 2SH-BTZ as marker of freshly generated climbing shoe abrasion. Similarly, in smaller halls (wall area: dbRDA 8% of variance, *Q*^2^ = 0.14) and halls with more visitors per wall area (5% of variance, univariate correlations), climbing activity is concentrated on fewer footholds, leading to continuous generation of fresh rubber particles. In these halls, 2SH-BTZ represented a higher fraction of rubber-derived compound composition in FP, while larger halls had higher fractions of 2NH-BTZ and HMMM. 2SH-BTZ also represented a higher fraction of rubber-derived compound composition in foothold powder in halls with shorter route duration, where footholds are changed more frequently and thus have fresher foothold powder, while 2NH-BTZ and HMMM represented higher fractions in halls which have longer route durations.

#### 3.3.3 Settled dust concentrations

34% of variation in rubber-derived compound concentrations in settled dust could be explained by climbing hall characteristics with RDA, slightly higher than for foothold powder. However, as for foothold powder, no single climbing hall characteristic explained more than 10% of variation in rubber-derived compound levels, with most climbing hall characteristics only accounting for a few percentage points of variation. The only climbing hall characteristic identified by more than one statistical method to influence rubber-derived compound concentrations in settled dust was visitors per wall area (9% of variation in dbRDA, *Q*^2^ = 0.43 in PLS). 2NH-BTZ and 2OH-BTZ had higher concentrations in more crowded halls, while BTZ and HMMM had lower concentrations in more crowded halls. When samples from cluster 4 were included, this trend remained significant and related hall characteristics wall surface area and weekly visitors were also identified as significant predictors of RDC concentrations in settled dust by our models. Additionally, when samples from cluster 4 were included, ventilation was identified by both RDA (6% of variation) and PLS-DA (BER_rel_ = 0.87) as a predictor of RDC concentrations in settled dust. This trend, not significant when cluster 4 samples were excluded, was driven by higher HMMM and 6PPDQ concentrations in halls without active ventilation.

#### 3.3.4 Settled dust compositions

Climbing hall characteristics explained 30% of the variation in the dbRDA of settled dust rubber-derived compound compositions. Only wall surface area (*Q*^2^ = 0.31 in PLS) and the related characteristic visitors per wall area (*Q*^2^ = 0.56 in PLS) were identified by PLS and had significant univariate relationships with three rubber-derived compounds: 2OH-BTZ represents a higher fraction in halls with lower wall surface area (and higher visitors per wall area), while HMMM and BTZ show the reverse trend. These trends were robust to inclusion or exclusion of samples from cluster 4 (SI.13.1), but the reasons behind them are unclear.

Overall, our results show that climbing hall characteristics do have some minor influence on the generation of rubber particles. In particular, hall age and chalk seem to impact the total concentrations of rubber-derived compounds detected in foothold powder, while hall type, wall surface area, visitors per wall area, and route duration were related to the rubber-derived compound profile, which depends on the foothold powder's age. Chalk influenced the rubber-derived compound composition, but the reason for the relationship needs further investigation and due to the low percentage of halls allowing only liquid chalk in our sample the result may not be reliable. Of all the climbing hall characteristics evaluated, only climbing wall surface area and visitors per wall area showed robust relationships with either total rubber-derived compound concentrations or rubber-derived compound composition in settled dust, and these trends were weak and not readily interpretable. Contrary to what we expected, chalk policy, mat type, ventilation, and route duration showed no relevant influence on the distribution of rubber-derived compounds through climbing hall air. However, both chalk policy and ventilation were unequally represented in our data, so further studies might find additional relationships.

## 4 Conclusions

Rubber-derived compounds (RDCs) were detected in all foothold powder and settled dust samples collected from 41 climbing halls, confirming that abrasion of climbing shoes consistently generates fine rubber particles that become airborne and subsequently settle as dust. Differences between compound profiles of foothold powder and settled dust samples reflect their differences in age and rubber particle morphology and fate, with greater transformations having occurred in the older settled dust samples. Across the ten hall characteristics examined, supervised analyses showed only weak relationships with RDC concentrations and compositions, indicating that hall characteristics have at most a minor influence compared to the chemical composition of climbing shoe soles. Eliminating health concerns associated with rubber-derived compound exposure in climbing halls will thus require the modification of rubber formulations to ensure that all climbing shoes are free of chemicals of concern.

This study is limited by the use of settled dust as a proxy for airborne particulate matter and by incomplete metadata for some facilities, which constrained statistical power for certain comparisons. Additionally, the chemically heterogeneous RDCs exhibit differences in recovery and limit of quantification, including between batches. Random substitution of <LOQ values should minimize bias in our results,^42,43,51^ but the effect of various substitution strategies was not evaluated in this study. Nonetheless, the multi-country sample set provides robust evidence that RDC contamination in climbing halls is widespread and largely insensitive to hall-specific features.

Given the modest influence of hall characteristics observed here, mitigation will be most effective at the material source. In line with international research on plastic additives and recommended approaches to guide the innovation process for chemicals and materials,^62^ consumer rubber products such as climbing shoe soles should be developed within a safe and sustainable-by-design (SSbD) framework that prioritizes substituting additives of concern with demonstrably safer alternatives and considers the formation of toxic transformation products under realistic use conditions.

Future work should couple direct measurements of airborne RDCs and personal exposure with material-level assessments of alternative formulations to resolve how design choices translate into indoor air concentrations and exposure in climbing halls and similar indoor environments.

## Author contributions

Sherman: conceptualization, methodology, software, validation, formal analysis, investigation, data curation, writing – original draft, writing – review & editing, visualization, supervision, project administration. Lotteraner: methodology, software, validation, formal analysis, data curation, writing – original draft, writing – review & editing, visualization. Maruschka: investigation, writing – review & editing, project administration. Hofmann: resources, writing – review & editing, supervision, funding acquisition.

## Conflicts of interest

There are no conflicts to declare.

## Acknowledgements

The authors would like to thank all volunteers who collected samples. The authors would also like to thank Tung Nguyen, Lukas Wimmer, and Thibault Masset for their help in the design of this study and along the way. Anya Sherman acknowledges funding from the University of Vienna Research Platform “Plenty”, Laura Lotteraner acknowledges funding from the University of Vienna Research Platform “Urban Futures”. Thilo Hofmann acknowledges funding from the Austrian Science Fund, Cluster of Excellence COE7, Grant DOI 10.55776/COE7.

## Supplementary Material

EM-028-D5EM00812C-s001

## Data Availability

Data for this article, including raw concentration data and survey data from all samples, as well as R code used for all statistical analyses are available at GitHub at github.com/Laura-Lotteraner/RDCs-Climbing-Halls. Supplementary information (SI) is available. See DOI: https://doi.org/10.1039/d5em00812c.
