# Supplementary material for: Minor influence of climbing hall characteristics on rubber-derived compound contamination highlights a need for material-level solutions
Source: Environ Sci Process Impacts. 2026 Jan 6;28(2):468–80. doi: 10.1039/d5em00812c (PMC12795585; doi:10.1039/d5em00812c)
Supplement: EM-028-D5EM00812C-s001 [file EM-028-D5EM00812C-s001.pdf]

Supplementary Information (SI) for Environmental Science: Processes & Impacts.
This journal is © The Royal Society of Chemistry 2026

Supplemental Information for

Minor influence of climbing hall characteristics on rubber-derived compound contamination highlights a need for material-level solutions

Anya Sherman[†,1], Laura Lotteraner[†,1,2,3], Leah K. Maruschka[1], Thilo Hofmann[1,*]

[†]These authors contributed equally.
[1]University of Vienna, Centre for Microbiology and Environmental Systems Science, Department of Environmental Geosciences, Josef-Holaubek-Platz 2, 1090 Vienna, Austria.
[2]University of Vienna, Faculty of Computer Science, Visualization and Data Analysis, 1090 Vienna, Austria.
[3]University of Vienna, Faculty of Computer Science, Doctoral School Computer Science, 1090 Vienna, Austria.
[*]Corresponding author.

SI.1 Data and Code 1
SI.2 Sampling Instructions 2
SI.3 Method blank contamination (ng/g equivalents) 7
SI.4 UPLC-MS/MS Method details 7
SI.5 Limits of quantification of all rubber-derived compounds 11
SI.6 Recovery of all rubber-derived compounds 40
SI.7 Summary statistics before and after random value substitution 41
SI.8 Clustering 43
SI.9 PLS(-DA) Implementation Details 43
SI.10 PLS(-DA) Results 45
SI.11 Univariate Correlations 48
SI.12 (db)RDA Results 57

SI.1 Data and Code

We provide our raw data and our code on https://github.com/Laura-Lotteraner/RDCs-

Climbing-Halls.

SI.2 Sampling Instructions

Climbing Hall Sampling Instructions

Contents of Kit:

- Metal spatula for sampling
- 6 paper tissues for cleaning metal spatula
- 6 pre-labelled sample collection vials
- 1 extra (non-labelled) sample collection vial

Foothold powder sample description:

Foothold powder is the accumulation of rubber particles which are formed due to the friction of climbing shoes with footholds. It represents a composite sample of different rubber from many different climbing shoes used within a climbing gym. See photo below.

Locate three sampling sites. Search for footholds with obvious accumulation of black powder. Search for holds that are primarily used as footholds (i.e. mostly accumulation of black powder rather than chalk). Material from several footholds, and from several routes can be combined into one sample, but they should all be within the same section of the gym. The three samples should be taken from different sections of the gym. For example, if the gym sets boulders on a schedule, each sample can represent a different set.

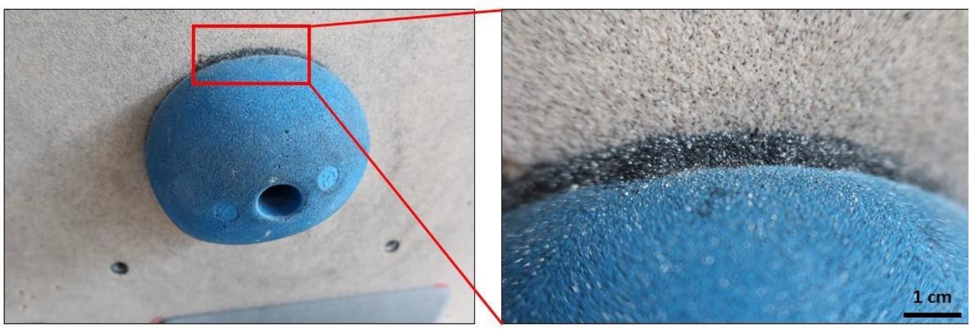

Settled dust sample description:

Settled dust represents particles which were once airborne and have settled out of the air. By measuring the chemical composition of settled dust, we can estimate the chemical composition of airborne particles which may be inhaled.

Locate three sampling sites. Search for areas in the climbing gym which are not frequently cleaned, where dust has visibly accumulated (examples – behind climbing walls, above climbing walls, behind Kilter/Moon boards). The important thing is that any rubber particles collected here must have been transported through the air before settling (so don't take samples directly under a climbing wall, where rubber particles could simply fall from the wall). See photo examples below:

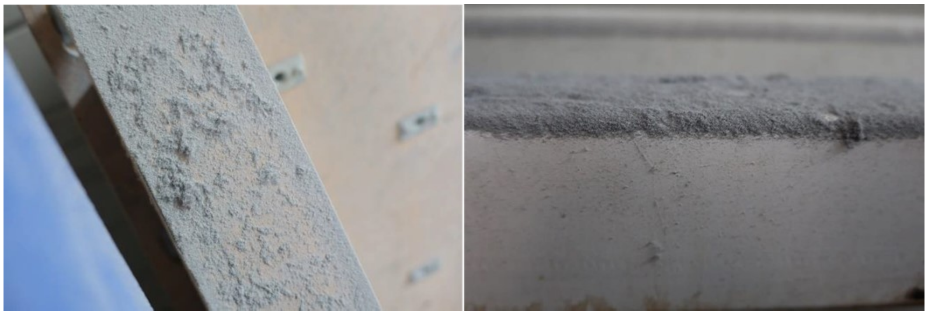

Sampling Instructions (settled dust and foothold powder):

1. Take a photo of each sampling site. Multiple photos may be necessary (for example, a close up and a zoomed out photo, to clearly illustrate the sampling site).
2. Wipe the metal spatula with a fresh paper tissue. Discard the tissue.
3. Use the spatula to scoop settled dust/foothold power into the collection vial. Fill until the marked line. You can use a clean brush to help in the collection if the amount of sample is limited.

    Note: 50 mg of sample is needed for the analysis. When sampling settled dust, take care to fill the sample vial with fine powder until the marked line. A single "hairball" of fiberous material will not suffice.
4. Tightly seal the collection vial.
5. Repeat for three settled dust and three foothold powder samples
6. If possible, wrap the vials in paper towel or tissue before repacking, to protect against breakage during shipping.
7. Fill out the attached form.

*If you make a mistake, one extra sampling vial is included. Label it with a permanent marker (keep the same label that was on the vial it should replace).

Return of samples:

Repackage the entire kit (6 sealed vials and metal spatula) in the envelope included in the kit. Return shipping instructions vary depending on country, and are included separately in your kit.

Photos should be emailed to the following email address. Photo files should either be named according to the label on the corresponding sample vial, or the photos should be clearly annotated with the label. Note: copying photos into the body of an email removes their name. Please make sure to send photos as attachments. If you would prefer, we can also provide an upload link upon request.

anya.sherman@univie.ac.at

The attached form may be either filled out electronically and returned via email, or by hand and returned via mail along with the samples.

Upon receiving all material, we will provide you with an estimated analysis time before we can send you results.

Contact Information of Sampler

Full Name ____

Email Address ____

Climbing Hall Information

Climbing Hall Name* _____________________________________

Climbing hall address*:

____

____

City, Country ____

Type of hall: ☐ Boulder ☐ Rope climbing ☐ Other (specify below)

____

Ventilation system (please describe the best you can, include information about windows/doors):

____

____

____

Chalk usage: ☐ All chalk ☐ Liquid chalk ☐ No chalk

Average number of visitors per week: ________________________

Type: ☐ Public ☐ Private (elite training facility) ☐ Private (non-elite)

☐ Other (describe below)

____

Square meters of gym (often on gym website): _______________

Volume of gym (if known): _________________

Wall surface area (if known, often on website): ____________________

Age of the gym: _________________________________

Average duration of time a route is up: __________________________

Mat type: ☐ Smooth surface ☐ Fiber surface ☐ No mats ☐ Other (specify below)

____

Wall type: ☐ no-texture ☐ friction (sand-coated or other) ☐ Wood ☐ Other (specify below)

____

Other comments:

____

____

____

____

____

____

*This information is for internal records only. The names of climbing halls will never be published or made available in any way.

SI.3 Method blank contamination (ng/g equivalents)

	6PPD	6PPDQ	IPPD	IPPDQ	CPPD	CPPDQ	DPPD	DPPDQ
Mean	0	0	0	0	0	0	0	0
SD	0	0	0	0	0	0	0	0
DF	0%	0%	0%	0%	0%	0%	0%	0%

	2SH-BTZ	BTZ	2OH-BTZ	2NH-BTZ	HMMM	DPG
Mean	0	0	0	0	0.31	5.1
SD	0	0	0	0	1.28	9.68
DF	0%	0%	0%	0%	6%	29%

Table SI.3.1: Summary statistics for compounds detected in method blanks (Mean, SD, Detection Frequency), in ng/g equivalents

SI.4 UPLC-MS/MS Method details

Compound Name	ISTD	Precursor (m/z)	Product (m/z)	Fragmentor Voltage (V)	Collision Energy (V)	Cell Accelerator Voltage (V)	RT (min)
2-aminobenzothiazole	d4-BTZ	151	109	150	30	5	5.4
2-aminobenzothiazole		151	65	150	38	5	5.4
2-hydroxybenzothiazole	d4-BTZ	152	124	140	22	4	3.6

Compound Name	ISTD	Precursor (m/z)	Product (m/z)	Fragmentor Voltage (V)	Collision Energy (V)	Cell Accelerator Voltage (V)	RT (min)
2-hydroxybenzothiazole		152	92	140	26	4	3.6
2-mercaptobenzothiazole	d4-BTZ	168	135	135	28	5	3.1
2-mercaptobenzothiazole		168	124	135	24	5	3.1
2-mercaptobenzothiazole		168	109	135	28	5	3.1
6PPD	d5-6PPD-q	269	184	150	45	5	3.8
6PPD		269	107	150	45	5	3.8
6PPD		269	93	150	45	5	3.8
6PPD-quinone		299	241	150	53	5	5.8
6PPD-quinone		299	215	150	30	5	5.8
6PPD-quinone	d5-6PPD-q	299	187	150	30	5	5.8
Aniline	d4-BTZ	94	77	100	22	4	2.6
Aniline		94	51	100	23	4	2.6
Aniline		94	50	100	41	4	2.6
Benzothiazole	d4-BTZ	136	109	150	31	5	2.8
Benzothiazole		136	77	150	27	5	2.8
Benzothiazole		136	65	150	38	5	2.8

Compound Name	ISTD	Precursor (m/z)	Product (m/z)	Fragmentor Voltage (V)	Collision Energy (V)	Cell Accelerator Voltage (V)	RT (min)
Benzothiazole-d4		140	113	150	31	5	4.5
Benzothiazole-d4		140	81	150	19	5	4.5
Benzothiazole-d4		140	69	150	36	5	4.5
CPPD	d5-6PPD-q	267.2	184	100	30	5	3.8
CPPD		267.2	107	100	50	5	3.8
CPPD		267.2	93.1	100	46	5	3.8
CPPD-quinone		297.1	215	130	18	5	6
CPPD-quinone		297.1	187	130	34	5	6
CPPD-quinone	d5-6PPD-q	297.1	55.2	130	50	5	6
d5-6PPD-quinone		304.2	246.1	110	36	4	6.5
d5-6PPD-quinone		304.2	220.1	110	36	5	6.5
d5-6PPD-quinone		304.2	192.1	110	36	5	6.5
DPG		212	195	150	20	5	1.8
DPG	d5-6PPD-q	212	119	150	20	5	1.8
DPG		212	94	150	20	5	1.8
DPPD		260.1	183	130	38	5	6.4

Compound Name	ISTD	Precursor (m/z)	Product (m/z)	Fragmentor Voltage (V)	Collision Energy (V)	Cell Accelerator Voltage (V)	RT (min)
DPPD	d5-6PPD-q	260.1	167	130	46	5	6.4
DPPD		260.1	156	130	46	5	6.4
DPPD-quinone	d5-6PPD-q	291.1	263	150	22	5	5.5
DPPD-quinone		291.1	235.2	150	34	5	5.5
DPPD-quinone		291.1	144	150	38	5	5.5
HMMM		391	283	150	15	5	3.5
HMMM		391	253	150	23	5	3.5
HMMM		391	207	150	19	5	3.5
HMMM	d5-6PPD-q	391	177	150	35	5	3.5
IPPD	d5-6PPD-q	227.2	184	100	18	5	3
IPPD		227.2	118	100	46	5	3
IPPD		227.2	107	100	46	5	3
IPPD-quinone		257.2	216	110	30	5	4.8
IPPD-quinone	d5-6PPD-q	257.2	187	110	30	5	4.8
IPPD-quinone		257.2	170	110	34	5	4.8

Table SI.4.1: Compound specific details for analysis with UPLC-MS/MS (Agilent 1290 Infinity II, Agilent 6470).

SI.5 Limits of quantification of all rubber-derived compounds

Batch	2NH-BTZ	DPG	2OH-BTZ	IPPD	BTZ	2SH-BTZ	HMMM	CPPD	6PPD	IPPDQ	DPPDQ	CPPDQ	DPPD
0	30	14.8	74	11.0	400	590.0	4	0.10	58.0	4	2.95	1.0	2.50
1	40	3.6	1000	0.4	1000	200.0	40	0.01	56.0	40	1.00	1.0	2.50
2	40	8.4	1000	4.0	400	100.0	4	0.10	40.0	40	1.0	1.0	2.50
3	40	10.8	1000	4.0	300	100.0	40	0.10	40.0	40	1.0	1.0	5.00
4	20	8.8	400	4.0	400	100.0	20	0.45	65.6	200	0.5	0.5	2.50
5	20	11.2	400	4.0	1000	964.8	20	0.10	25.2	100	1.0	0.5	3.38

Table SI.5.1: Limit of quantification for each batch (units in ng/g). Limit of quantification varied by batch due to variations in instrument sensitivity over time. Random substitution of <LOQ values was performed by batch to account for variable LOQs.

SI.6 Recovery of all rubber-derived compounds

Compound	Relative Recovery (%)
6PPD	87 ± 10
6PPDQ	97 ± 6
IPPD	90 ± 9
IPPDQ	117 ± 18
CPPD	101 ± 7
CPPDQ	109 ± 9
DPPD	132 ± 7
DPPDQ	74 ± 21
2SH-BTZ	32 ± 14
BTZ	98 ± 4
2OH-BTZ	89 ± 5
2NH-BTZ	98 ± 4
HMMM	145 ± 34
DPG	76 ± 20

Table SI.6.1: Relative Recovery of Compounds

SI.7 Summary statistics before and after random value substitution

Compound	DF	Type	Mean	25%	50%	75%
6PPD	99%	*raw*	4500 ng/g	577 ng/g	1900 ng/g	4700 ng/g
		sub	4400 ng/g	561 ng/g	1800 ng/g	4700 ng/g
6PPDQ	95%	*raw*	284 ng/g	126 ng/g	214 ng/g	308 ng/g
		sub	270 ng/g	117 ng/g	206 ng/g	306 ng/g
IPPD	100%	*raw*	117 ng/g	1300 ng/g	370 ng/g	1100 ng/g
		sub	117 ng/g	1300 ng/g	370 ng/g	1100 ng/g
IPPDQ	59%	*raw*	227 ng/g	44.2 ng/g	83.5 ng/g	205 ng/g
		sub	146 ng/g	22.8 ng/g	43.4 ng/g	109 ng/g
CPPD	70%	*raw*	22.5 ng/g	8.8 ng/g	12.0 ng/g	20.0 ng/g
		sub				
CPPDQ	0%	*raw*				
		sub				
DPPD	24%	*raw*	99.0 ng/g	12.0 ng/g	109 ng/g	115 ng/g
		sub				
DPPDQ	0%	*raw*				
		sub				
2SH-BTZ	92%	*raw*	117000 ng/g	47300 ng/g	89300 ng/g	141000 ng/g
		sub	107000 ng/g	38200 ng/g	78700 ng/g	132000 ng/g
BTZ	95%	*raw*	18400 ng/g	9600 ng/g	14100 ng/g	19600 ng/g
		sub	17900 ng/g	9300 ng/g	13900 ng/g	19100 ng/g
2OH-BTZ	91%	*raw*	18800 ng/g	11700 ng/g	16200 ng/g	24800 ng/g
		sub	17100 ng/g	9700 ng/g	15100 ng/g	24100 ng/g
2NH-BTZ	97%	*raw*	266 ng/g	144 ng/g	204 ng/g	305 ng/g
		sub	260 ng/g	137 ng/g	202 ng/g	301 ng/g
HMMM	90%	*raw*	4100 ng/g	51.5 ng/g	92.5 ng/g	242.2 ng/g
		sub	3700 ng/g	39.0 ng/g	81.1 ng/g	227.0 ng/g
DPG	96%	*raw*	35000 ng/g	12600 ng/g	23500 ng/g	46400 ng/g
		sub	33400 ng/g	11800 ng/g	21900 ng/g	44300 ng/g

Table SI.7.1: Summary statistics of compound concentrations in foothold powder, excluding outliers, before and after random value substitution for <LOQ samples. CPPD, CPPDQ, DPPD, and DPPDQ were excluded from all analyses, thus no substitution was performed.

Compound	DF	Type	Mean	25%	50%	75%
6PPD	60%	*raw*	101.7 ng/g	68.2 ng/g	81.0 ng/g	108 ng/g
		sub	72.9 ng/g	29.6 ng/g	60.9 ng/g	88.6 ng/g
6PPDQ	43%	*raw*	250 ng/g	68.0 ng/g	171 ng/g	311 ng/g
		sub	116 ng/g	7.39 ng/g	25.6 ng/g	139 ng/g
IPPD	90%	*raw*	27.5 ng/g	10.7 ng/g	14.0 ng/g	22.6 ng/g
		sub	24.8 ng/g	8.9 ng/g	13.0 ng/g	20.9 ng/g
IPPDQ	5%	*raw*	14.5 ng/g	2.49 ng/g	7.69 ng/g	23.6 ng/g
		sub				
CPPD	7%	*raw*	5.96 ng/g	4.56 ng/g	5.60 ng/g	6.54 ng/g
		sub				
CPPDQ	0%	*raw*				
		sub				
DPPD	3%	*raw*	35.6 ng/g	0.6 ng/g	0.8 ng/g	53.2 ng/g
		sub				
DPPDQ	3%	*raw*	56.4 ng/g	42.0 ng/g	46.8 ng/g	66.0 ng/g
		sub				
2SH-BTZ	8%	*raw*	10600 ng/g	627 ng/g	1600 ng/g	4200 ng/g
		sub				
BTZ	40%	*raw*	3790 ng/g	1150 ng/g	1930 ng/g	2990 ng/g
		sub	1700 ng/g	237 ng/g	432 ng/g	1520 ng/g
2OH-BTZ	65%	*raw*	2350 ng/g	1240 ng/g	1580 ng/g	2480 ng/g
		sub	1690 ng/g	661 ng/g	1200 ng/g	1920 ng/g
2NH-BTZ	56%	*raw*	78.0 ng/g	43.7 ng/g	56.6 ng/g	83.4 ng/g
		sub	51 ng/g	19 ng/g	40 ng/g	60 ng/g
HMMM	93%	*raw*	703 ng/g	55.7 ng/g	117 ng/g	223 ng/g
		sub	655 ng/g	49.2 ng/g	110 ng/g	198 ng/g
DPG	100%	*raw*	1070 ng/g	188 ng/g	417 ng/g	891 ng/g
		sub	1070 ng/g	188 ng/g	417 ng/g	891 ng/g

Table SI.7.2: Summary statistics of compound concentrations in settled dust, excluding outliers, before and after random value substitution for <LOQ samples. CPPD, CPPDQ, DPPD, DPPDQ, IPPDQ and 2SH-BTZ were excluded from all analyses, thus no substitution was performed.

SI.8 Clustering

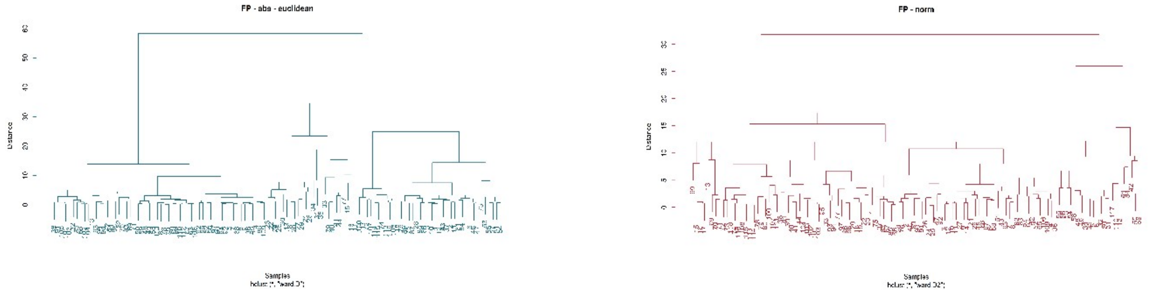

Figure SI.8.1: Dendrograms for hierarchical clustering with Euclidean (concentrations, blue) and Aitchison (compositions, red) distance of foothold powder samples.

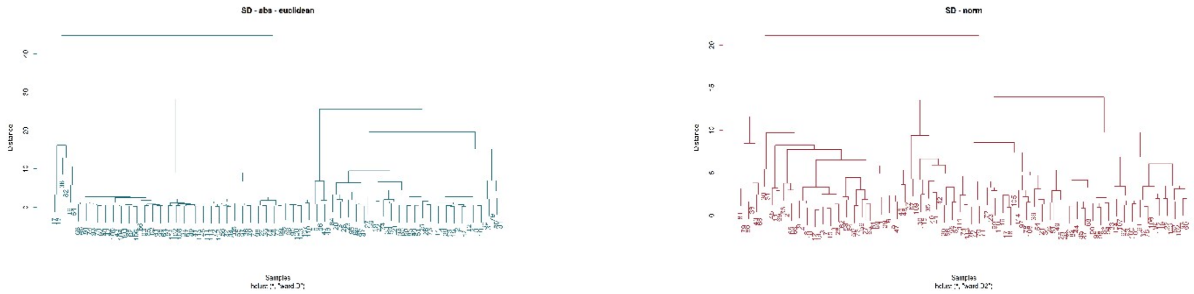

Figure SI.8.2: Dendrograms for hierarchical clustering with Euclidean (concentrations, blue) and Aitchison (compositions, red) distance of settled dust samples.

SI.9 PLS(-DA) Implementation Details

For both PLS and PLS-DA models, the datasets (foothold powder concentrations, settled dust concentrations, foothold powder composition, settled dust composition) were split such that two replicates from each hall were included in a training set, and the third was included in a test set. To compute Q^2 (PLS) and relative balanced error rate (BER) (PLS-DA), the descriptors of samples in the test dataset were predicted and compared to the actual sample descriptors. The balanced error rate BER assesses prediction accuracy across all classes

while considering uneven class sizes.

The full procedure can be found in the attached code (code.R).

SI.10 PLS(-DA) Results

	Hall Type	Chalk	Mat Type	Wall Type	Ventilation
**BER_{rel}**	**0.70**	**0.88**	**0.84**	1.02	1.02
6PPD	0.66	**1.49**	**1.02**	0.90	0.31
6PPDQ	0.53	**1.25**	0.71	1.05	0.95
IPPD	0.87	**1.83**	**1.19**	0.97	0.04
IPPDQ	**1.37**	0.51	0.38	0.94	1.94
2SH-BTZ	**1.79**	0.59	**1.07**	0.97	0.04
BTZ	0.73	0.59	**1.14**	1.01	10.03
2OH-BTZ	**1.09**	0.58	**1.07**	1.05	1.32
2NH-BTZ	0.78	0.52	**1.11**	1.25	1.33
HMMM	0.42	0.90	0.74	0.81	0.60
DPG	0.98	0.70	**1.22**	0.98	1.16

Table SI.10.1: **Partial least squares discriminant analysis (PLS-DA) on single categorical climbing hall characteristics versus foothold powder RDC concentrations**. Relative Balanced Error Rate (BER_{rel}) score for each characteristic indicates how much better than random the resulting PLS-DA model could predict the class of a test dataset, taking into account uneven class distribution (>1: worse than random, 1: random, 0: perfect prediction). For each compound, the variable importance in projection (VIP) scores are presented, where a VIP score greater than 1 indicates that this compound differed substantially between classes.

	Hall Age	Weekly Visitors	Wall Surface Area	Route Duration	Visitors / Wall Area
**Q^2**	**0.16**	-0.01	**0.11**	**0.13**	-0.05
6PPD	0.96	1.69	0.39	0.35	0.06
6PPDQ	0.74	0.19	0.65	0.03	0.10
IPPD	0.66	0.53	0.88	0.79	0.71
IPPDQ	0.26	0.95	**1.34**	0.69	1.70
2SH-BTZ	0.00	1.63	**1.86**	**1.82**	1.35
BTZ	0.81	0.31	0.50	0.86	0.88
2OH-BTZ	**2.20**	0.37	**1.36**	**1.10**	1.22
2NH-BTZ	**1.52**	0.41	0.93	0.86	0.71
HMMM	0.13	1.61	0.19	**1.56**	1.37
DPG	0.48	0.55	0.63	0.56	0.38

Table SI.10.2: **Partial Least Squares (PLS) regression on single quantitative climbing hall characteristics versus foothold powder RDC concentrations.** Q^2 indicates model performance on a test dataset (1: perfect prediction, ≤ 0: no predictive ability). For each compound, the variable importance in projection (VIP) scores are presented, where a VIP score greater than 1 indicates an important contribution to the PLS model.

	Hall Type	Chalk	Mat Type	Wall Type	Ventilation
**BER_{rel}**	0.98	1.15	0.99	1.03	1.00
6PPD	0.55	0.08	1.52	0.40	0.44
6PPDQ	1.06	1.30	0.64	1.79	1.66
IPPD	1.59	1.76	1.34	1.05	1.18
BTZ	0.73	0.41	1.02	0.70	0.66
2OH-BTZ	0.82	0.61	0.40	1.11	0.86
2NH-BTZ	1.51	0.41	1.02	1.02	0.64
HMMM	0.41	1.57	0.42	0.50	1.16
DPG	0.63	0.04	1.03	0.73	0.86

Table SI.10.3: **Partial least squares discriminant analysis (PLS-DA) on single categorical climbing hall characteristics versus settled dust RDC concentrations**. Relative Balanced Error Rate (BER_{rel}) score for each characteristic indicates how much better than random the resulting PLS-DA model could predict the class of a test dataset, taking into account uneven class distribution (>1: worse than random, 1: random, 0: perfect prediction). For each compound, the variable importance in projection (VIP) scores are presented, where a VIP score greater than 1 indicates that this compound differed substantially between classes.

	Hall Age	Weekly Visitors	Wall Surface Area	Route Duration	Visitors / Wall Area
**Q^2**	-0.06	**0.22**	**0.16**	**0.17**	**0.43**
6PPD	1.21	0.79	0.20	0.16	0.17
6PPDQ	1.32	**1.92**	0.85	**1.39**	0.07
IPPD	1.34	0.22	0.53	0.18	0.06
BTZ	0.56	0.15	**1.17**	**1.04**	**1.12**
2OH-BTZ	0.32	0.51	**1.35**	0.02	**1.00**
2NH-BTZ	0.33	0.37	1.37	0.90	**1.57**
HMMM	1.20	0.14	**1.20**	**1.79**	**1.64**
DPG	1.02	**1.79**	0.68	0.95	0.71

Table SI.10.4: **Partial Least Squares (PLS) regression on single quantitative climbing hall characteristics versus settled dust RDC concentrations.** Q^2 indicates model performance on a test dataset (1: perfect prediction, ≤ 0: no predictive ability). For each compound, the variable importance in projection (VIP) scores are presented, where a VIP score greater than 1 indicates an important contribution to the PLS model.

	Hall Type	Chalk	Mat Type	Wall Type	Ventilation
**BER_{rel}**	**0.62**	**0.51**	**0.76**	1.01	1.03

Table SI.10.5: **Partial least squares discriminant analysis (PLS-DA) on single categorical climbing hall characteristics versus foothold powder RDC composition**. Relative Balanced Error Rate (BER_{rel}) score for each characteristic indicates how much better than random the resulting PLS-DA model could predict the class of a test dataset, taking into account uneven class distribution (>1: worse than random, 1: random, 0: perfect prediction). Since PLS-DA was performed on ILR-transformed compositional data, components do not directly correspond to rubber-derived compounds, and no VIP scores are provided.

	Hall Age	Weekly Visitors	Wall Surface Area	Route Duration	Visitors / Wall Area
**Q^2**	-0.02	0.09	**0.14**	**0.17**	-0.27

Table SI.10.6: **Partial Least Squares (PLS) regression on single quantitative climbing hall characteristics versus foothold powder RDC compositions.** Q^2 indicates model performance on a test dataset (1: perfect prediction, ≤ 0: no predictive ability). Since PLS- DA was performed on ILR-transformed compositional data, components do not directly correspond to rubber-derived compounds, and no VIP scores are provided.

	Hall Type	Chalk	Mat Type	Wall Type	Ventilation
**BER_{rel}**	**0.70**	1.05	0.94	1.04	1.03

Table SI.10.7: **Partial least squares discriminant analysis (PLS-DA) on single categorical climbing hall characteristics versus settled dust RDC composition**. Relative Balanced Error Rate (BER_{rel}) score for each characteristic indicates how much better than random the resulting PLS-DA model could predict the class of a test dataset, taking into account uneven class distribution (>1: worse than random, 1: random, 0: perfect prediction). Since PLS-DA was performed on ILR-transformed compositional data, components do not directly correspond to rubber-derived compounds, and no VIP scores are provided.

	Hall Age	Weekly Visitors	Wall Surface Area	Route Duration	Visitors / Wall Area
**Q^2**	-0.13	**0.25**	**0.31**	**0.28**	**0.56**

Table SI.10.8: **Partial Least Squares (PLS) regression on single quantitative climbing hall characteristics versus settled dust RDC compositions.** Q^2 indicates model performance on a test dataset (1: perfect prediction, ≤ 0: no predictive ability). Since PLS- DA was performed on ILR-transformed compositional data, components do not directly correspond to rubber-derived compounds, and no VIP scores are provided.

SI.11 Univariate Correlations

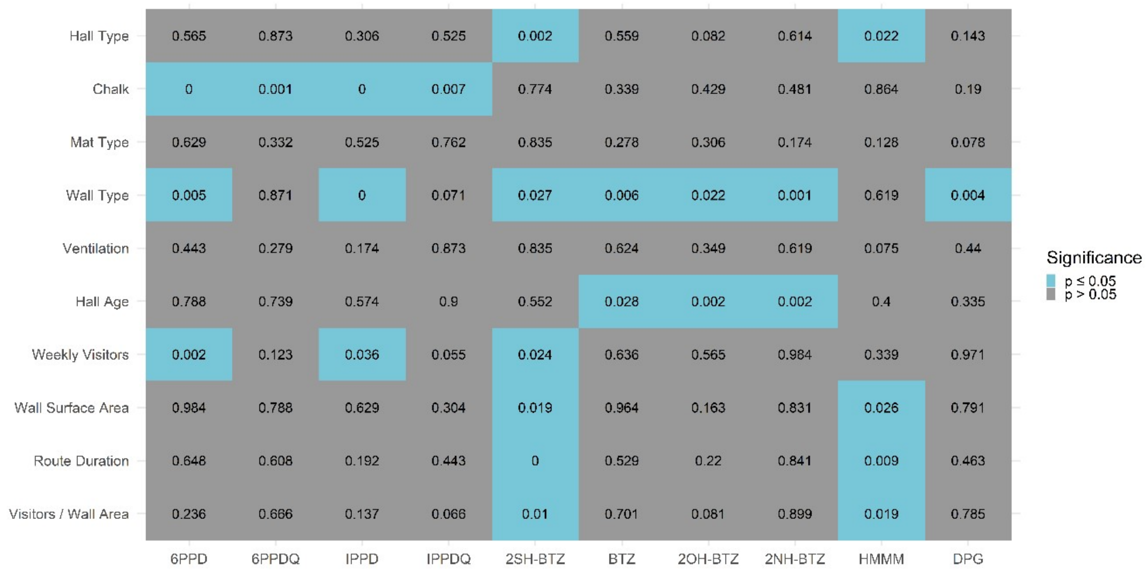

Figure SI.11.1: Univariate correlations between climbing hall characteristics and RDC concentrations in foothold powder

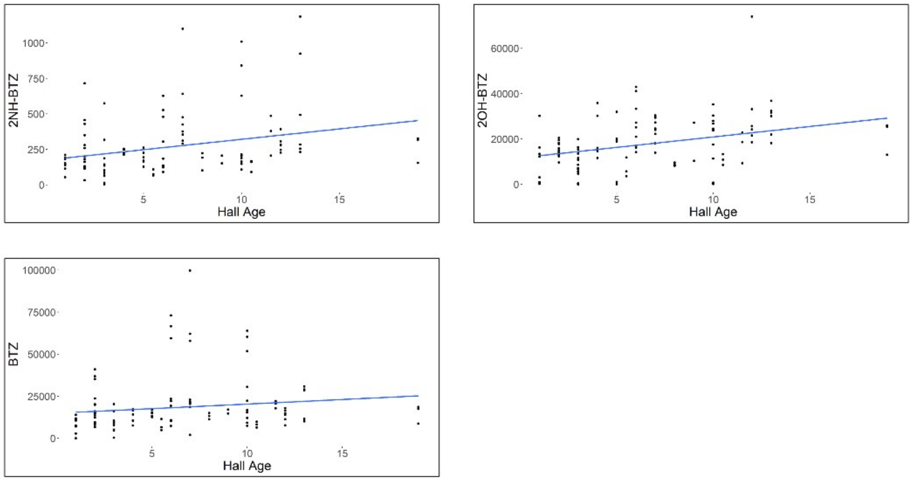

Figure SI.11.2: Significant relationships between hall age and individual compound concentrations in foothold powder.

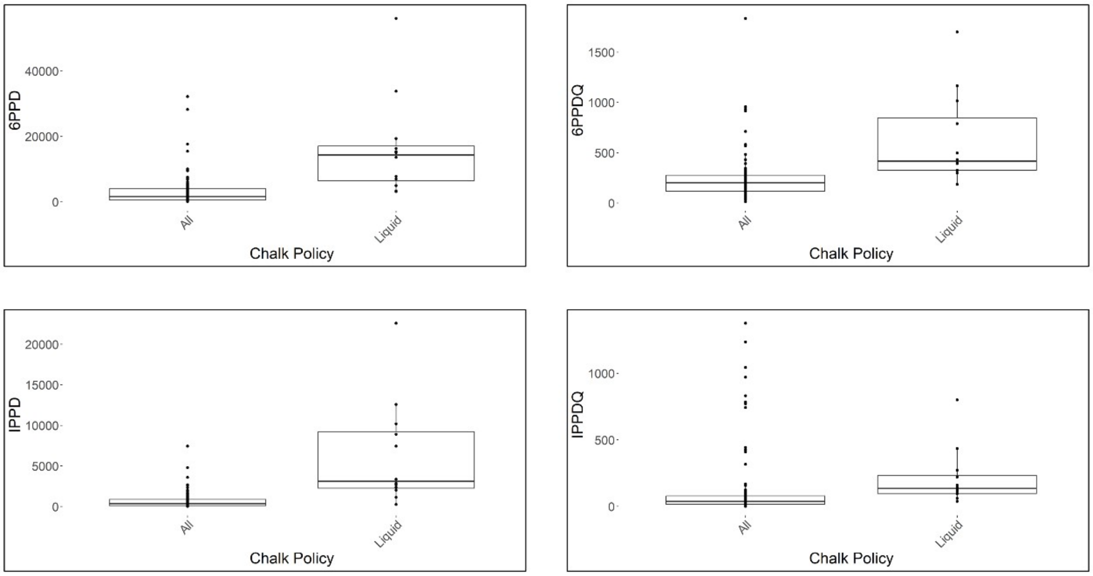

Figure SI.11.3: Significant relationships between chalk policy and individual compound concentrations in foothold powder.

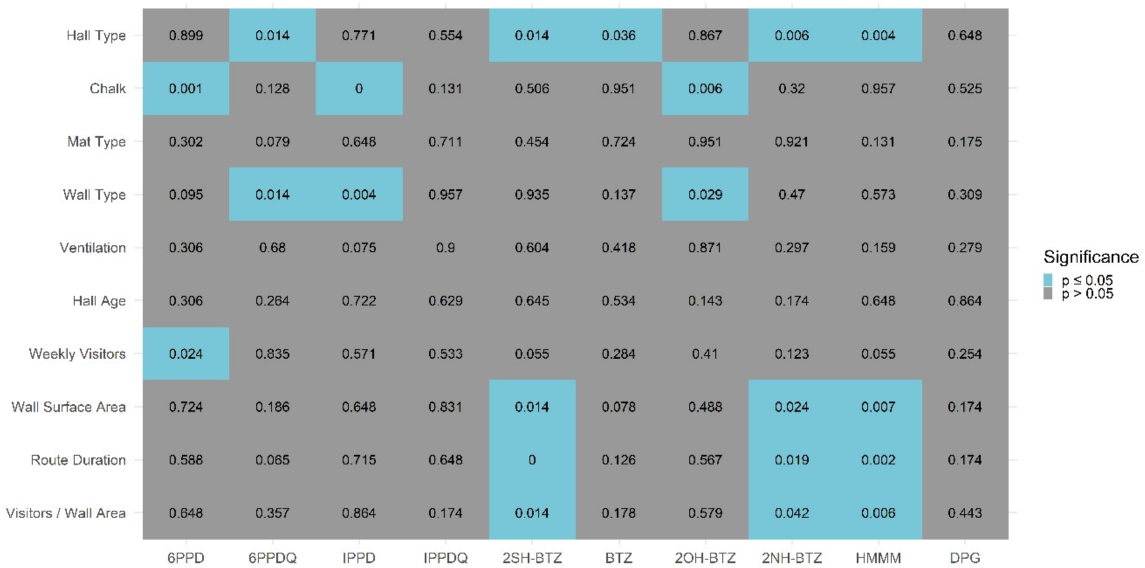

Figure SI.11.4: Univariate correlations between climbing hall characteristics and RDC composition in foothold powder

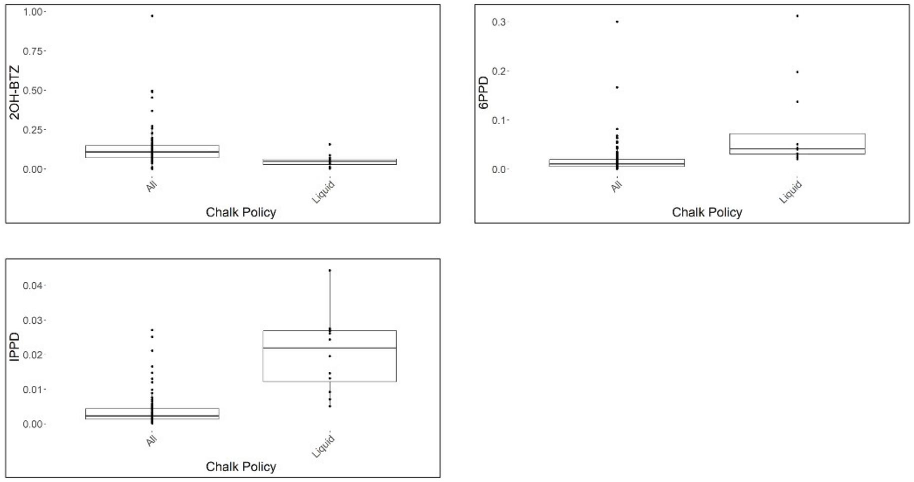

Figure SI.11.5: Significant relationships between chalk policy and individual compound compositions in foothold powder.

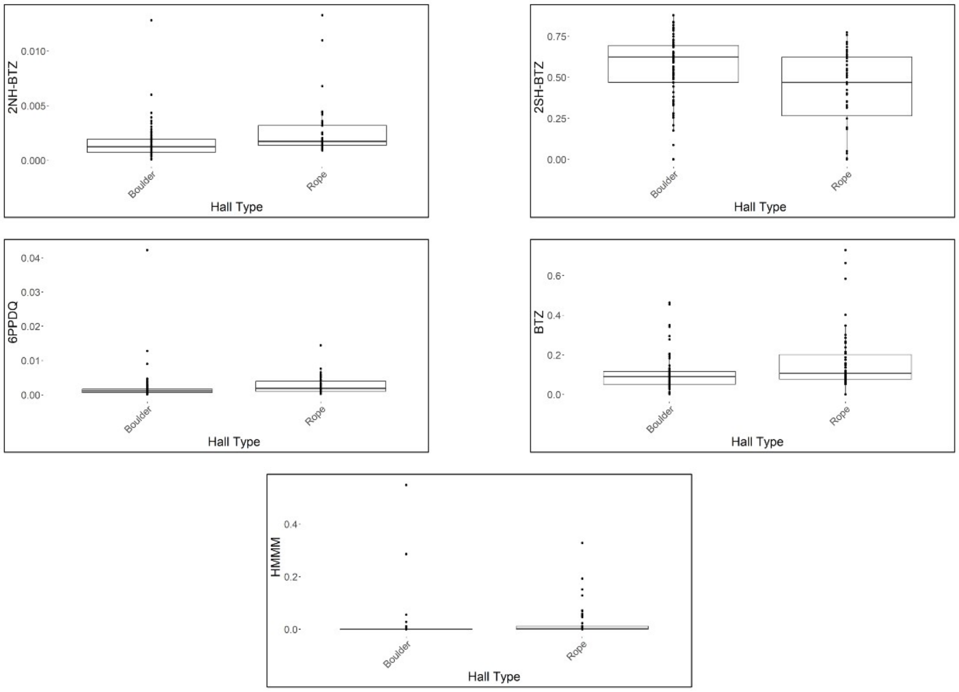

Figure SI.11.6: Significant relationships between hall type and individual compound compositions in foothold powder.

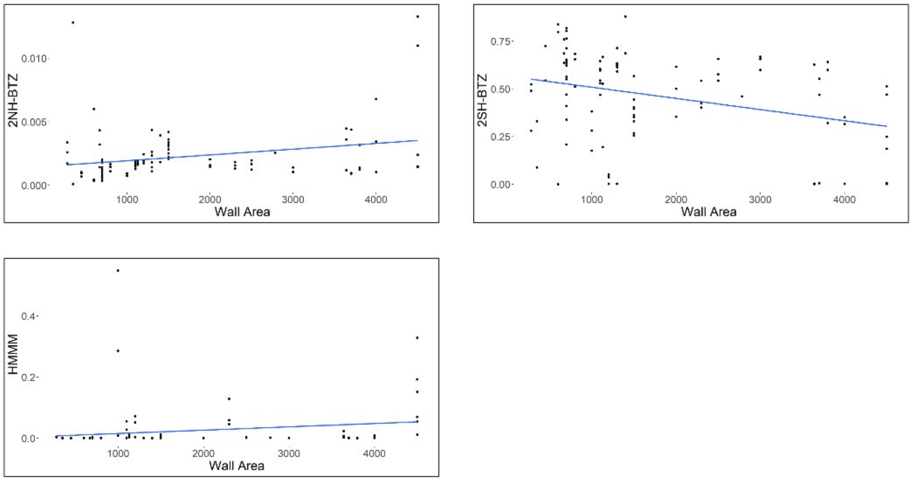

Figure SI.11.7: Significant relationships between wall area and individual compound compositions in foothold powder.

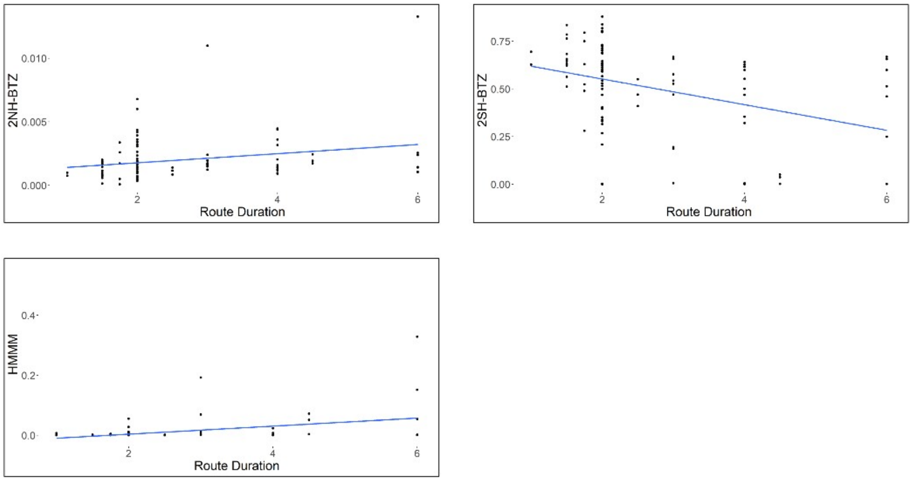

Figure SI.11.8: Significant relationships between route duration and individual compound compositions in foothold powder.

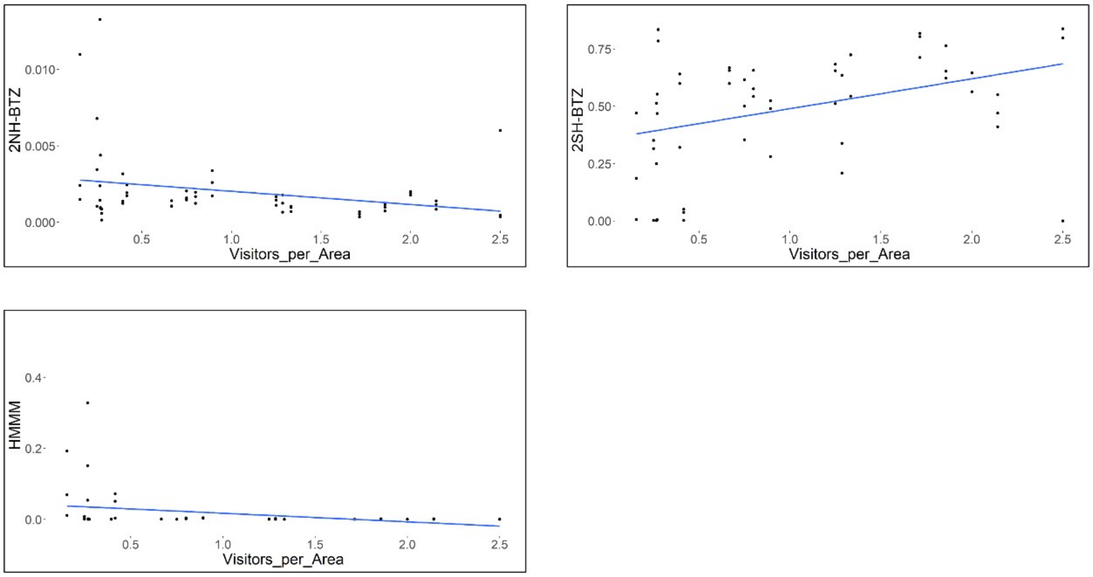

Figure SI.11.9: Significant relationships between visitors per wall area and individual compound compositions in foothold powder.

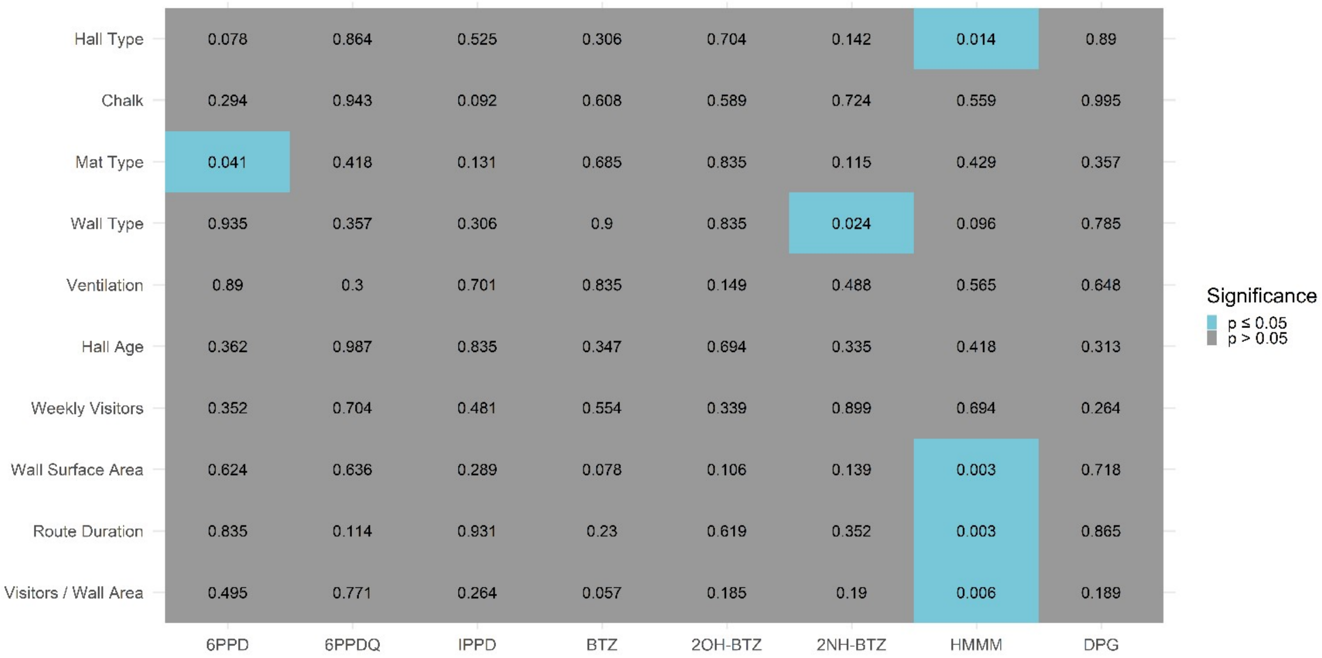

Figure SI.11.10: Univariate correlations between climbing hall characteristics and RDC concentrations in settled dust

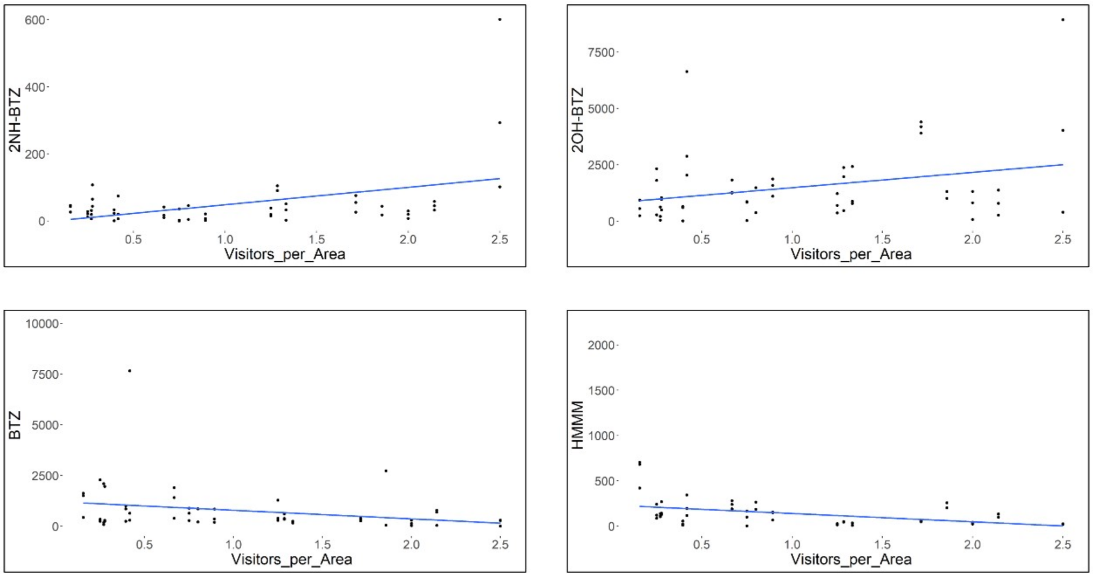

Figure SI.11.11: Significant relationship between visitors per wall area and individual compound concentrations in settled dust.

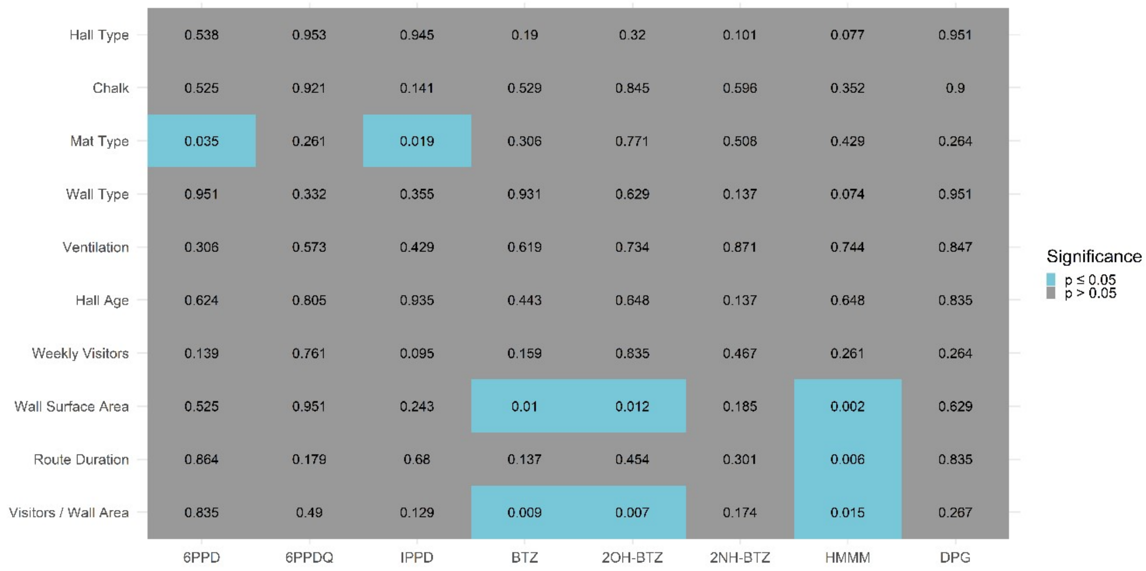

Figure SI.11.12: Univariate correlations between climbing hall characteristics and RDC composition in settled dust

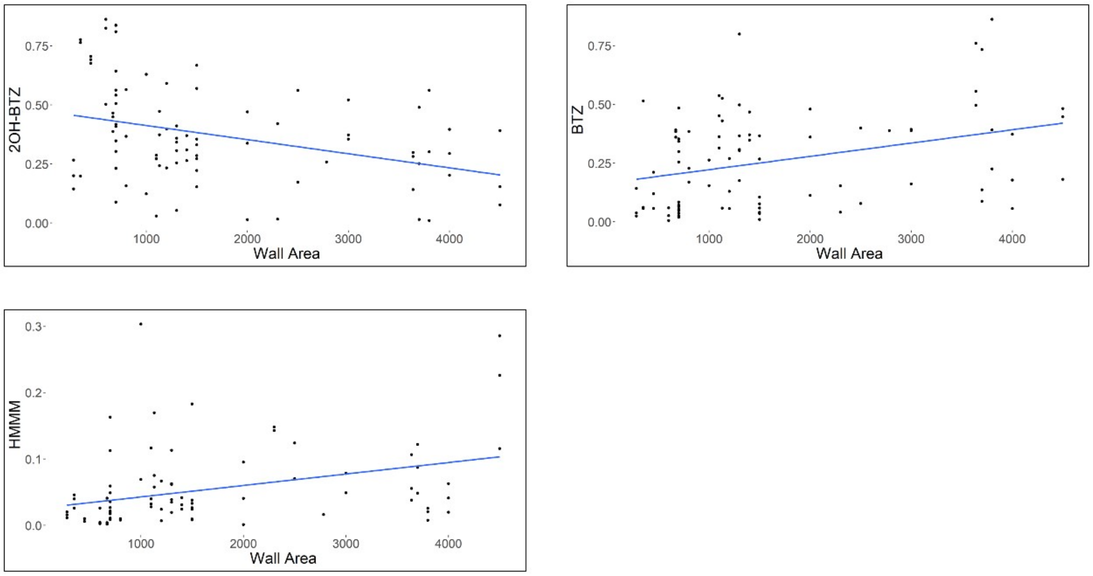

Figure SI.11.13: Significant relationships between wall area and individual compound compositions in settled dust.

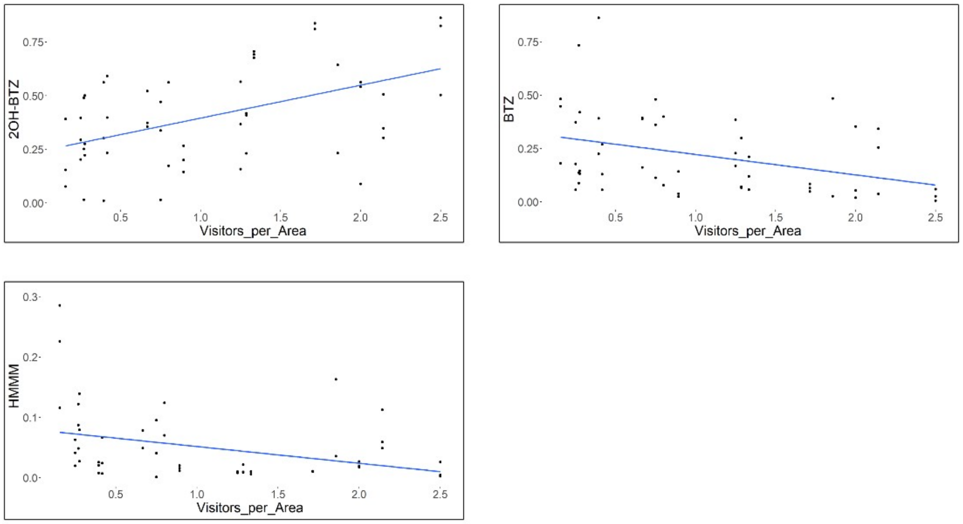

Figure SI.11.14: Significant relationships between visitors per area and individual compound compositions in settled dust.

SI.12 (db)RDA Results

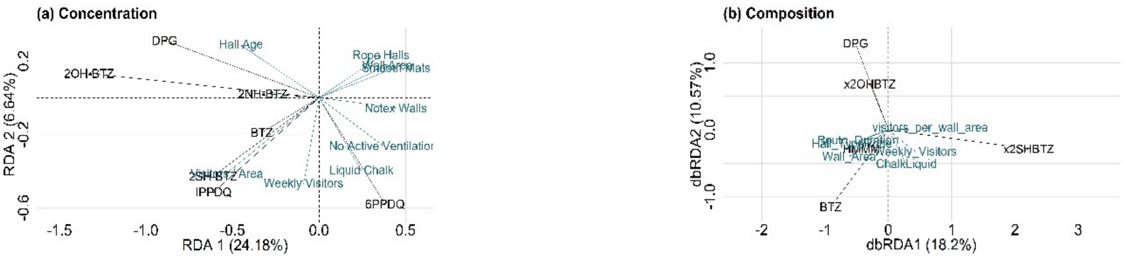

Figure SI.12.1: First and second component of RDA (concentration) and dbRDA (compositions) of foothold powder samples.

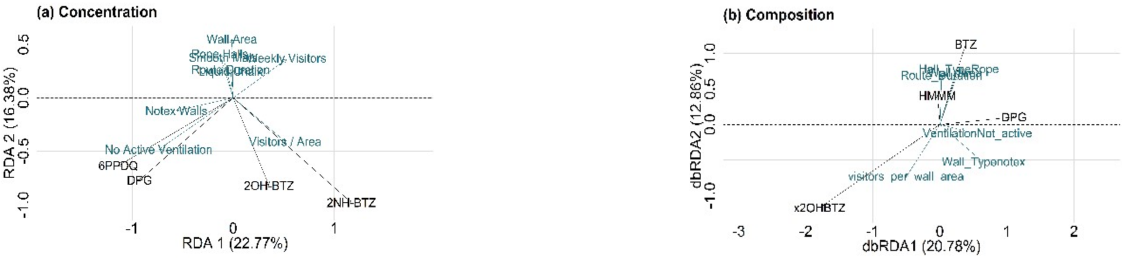

Figure SI.12.2: First and second component of RDA (concentration) and dbRDA (compositions) of settled dust samples.

	Foothold Powder		Settled Dust	
	Concentrations	Compositions	Concentrations	Compositions
**Total Variance (adjusted)**	**26%**	**24%**	**38%**	**30%**
Weekly Visitors	3%	**6%**	3%	1%
Wall Surface Area	3%	**8%**	**4%**	2%
Visitors / Wall Area	1%	**5%**	9%	2%
Wall Type	4%	1%	1%	**5%**
Ventilation	3%	1%	**7%**	3%
Route Duration	1%	2%	0%	0%
Mat Type	4%	1%	1%	4%
Hall Type	0%	4%	**6%**	1%
Hall Age	**5%**	1%	3%	1%
Chalk	3%	**5%**	3%	2%

Table SI.12.1: Variance in RDC concentrations and compositions explained by climbing hall characteristics according to redundancy analysis (concentrations) and distance-based redundancy analysis (compositions). *Variance for individual climbing hall characteristics* is their marginal contribution, i.e., the amount of variation that can be explained by each characteristic alone, without accounting for interaction effects with other characteristics. *Total adjusted variance* is the total variance that can be explained by the combination of all climbing hall characteristics, accounting for interaction effects between characteristics and model complexity. Due to their differences in calculation and meaning, the total adjusted variance does not necessarily equal the sum of the variances of the individual climbing hall characteristics.

SI.13 Sensitivity Analysis: Cluster 4 Inclusion

	**Foothold Powder**		**Settled Dust**	
	Concentrations	Compositions	Concentrations	Compositions
**Chalk Policy**	**PLS-DA (0.81)**<br>**Univariate (4)**	**dbRDA (0.05)**<br>**PLS-DA (0.51)**<br>**Univariate (3)**		
**Hall Age**	**RDA (0.05)**<br>**PLS (0.16)**<br>**Univariate (3)**		PLS (0.25)	
**Hall Type**	PLS-DA (0.70)	**PLS-DA (0.57)**<br>**Univariate (5)**	RDA (0.06)	PLS-DA (0.74)
**Route Duration**	PLS (0.13)	**PLS (0.17)**<br>**Univariate (3)**		PLS (0.36)
**Visitors / Wall Area**		**dbRDA (0.05)**<br>**Univariate (3)**	**RDA (0.09)**<br>**PLS (0.36)**	**PLS (0.57)**<br>**Univariate (3)**
**Wall Surface Area**	PLS (0.11)	**dbRDA (0.08)**<br>**PLS (0.14)**<br>**Univariate (3)**	**RDA (0.07)**<br>**PLS (0.12)**	**dbRDA (0.06)**<br>**PLS (0.36)**
**Weekly Visitors**	Univariate (3)	dbRDA (0.06)	**RDA (0.05)**<br>**PLS (0.23)**	PLS (0.22)
**Ventilation**			**RDA (0.06)**<br>**PLS-DA (0.87)**	
Mat Type	PLS-DA (0.85)		PLS-DA (0.86)	PLS-DA (0.89)
Wall Type	Univariate (6)	Univariate (3)		dbRDA (0.05)

Table SI.13.1: Combined results of (db)RDA, PLS(-DA) and univariate correlations for foothold powder and settled dust concentrations and compositions with Cluster 4 samples included. Numbers in brackets are the marginal contribution to the overall variation for RDA and dbRDA, Q^2 for PLS models, BER_{rel} for PLS-DA models, and the number of compounds with a significant relationship in the univariate case. Hall characteristics selected by at least two different models and the corresponding model scores are highlighted in bold.
